# DNA barcoding reveals the temporal community composition of drifting fish eggs in the lower Hongshui River, China

**DOI:** 10.1002/ece3.7943

**Published:** 2021-07-22

**Authors:** Weitao Chen, Shuli Zhu, Jiping Yang, Xinhui Li, Yuefei Li, Jie Li

**Affiliations:** ^1^ Pearl River Fisheries Research Institute Chinese Academy of Fishery Science Guangzhou China; ^2^ Key Laboratory of Aquatic Animal Immune Technology of Guangdong Province Guangzhou China; ^3^ Guangzhou Scientific Observing and Experimental Station of National Fisheries Resources and Environment Guangzhou China; ^4^ Experimental Station for Scientific Observation on Fishery Resources and Environment in the Middle and Lower Reaches of Pearl River Zhaoqing China

**Keywords:** DNA barcodes, fish eggs, lower Hongshuihe River, spawning periods, species composition

## Abstract

Determining the temporal community composition of fish eggs in particular regions and understanding the reproductive times of regional fish taxa are key aspects of the management and regulation of regional fish stocks. However, it is extremely difficult to accurately identify fish eggs due to the absence of diagnostic morphological characters. We sampled fish eggs in the lower Hongshuihe River (an upper mainstem of the Pearl River) between May and September 2020. We then used DNA barcoding to determine the species composition of the egg pool and to predict the spawning periods of the identified species. A total of 641 eggs and 17 larvae were chosen for molecular identification; 397 eggs and 17 larvae yielded high‐quality barcoding sequences. The high failure rate (~38%) was most likely due to long‐term storage in low concentrations of ethanol prior to molecular analysis. We successfully classified 392 eggs into 10 species and 13 larvae into four species using public databases. Most of the species identified in the egg pool were small and/or benthic, and migratory species were rare. This may partially reflect the adverse effects of hydropower cascade development in this river section. We also found that spawning periods tended to be species‐specific. Our study provides a reference for the conservation and management of regional fishery stocks.

## Graphical abstract

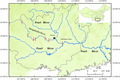

## 1 INTRODUCTION

Fish eggs and larvae represent important sources of information about the early phases of fish development, providing valuable data about spawning locations, spawning times, recruitment success rates, and the reproductive intensities of multiple species (Baumgartner et al., 2004; Bialetzki et al., 2005; Cao et al., 2007). An accurate understanding of this information has important implications for the management of regional fish resources (Armsworth, 2002; Liu et al., 2018). Fish eggs provide more direct data concerning the abundance of spawning fish populations than larvae due to the reduced cumulative influence of egg transport and mortality (Richardson et al., 2009). However, species‐level information about the composition and reproductive timing of the egg pool remains very limited, mostly because fish eggs have limited visible traits that can be used for identification. High morphological and size similarities among the eggs of different species, as well as the drastic ontogenetic changes that occur even within a single species, render the accurate species‐level identification of fish eggs exceedingly difficult (Burghart et al., 2014; Cao et al., 2007; Gao et al., 2010; Hofmann et al., 2017). To accurately classify fish into species, researchers typically raise eggs to a juvenile or adult stage that has diagnostic morphological traits (Cao et al., 2007; Gao, 2018; Gao et al., 2019; Xu et al., 2015). However, it is difficult to hatch eggs in the field, and larvae have high mortality rates during the feeding stage.

DNA barcoding, which delimits species based on a segment of the mitochondrial cytochrome *c* oxidase subunit I (COI), has been widely used to identify fish eggs (Chen et al., 2021; Hofmann et al., 2017; Hou et al., 2020; Kerr & Breitbart, 2021; Kerr et al., 2020; Leyva‐Cruz et al., 2016). For example, Leyva‐Cruz et al. (2016) collected 300 fish eggs from waters surrounding Banco Chinchorro in the Mexican Caribbean and assigned the eggs to 42 species using DNA barcoding. Similarly, Kerr et al. (2020) used DNA barcoding to identify fish eggs collected from northwestern Cuba and across the Florida Straits, assigning 564 fish eggs to 89 species. Therefore, it has been demonstrated that DNA barcoding can effectively identity fish eggs to species.

**FIGURE 1:**
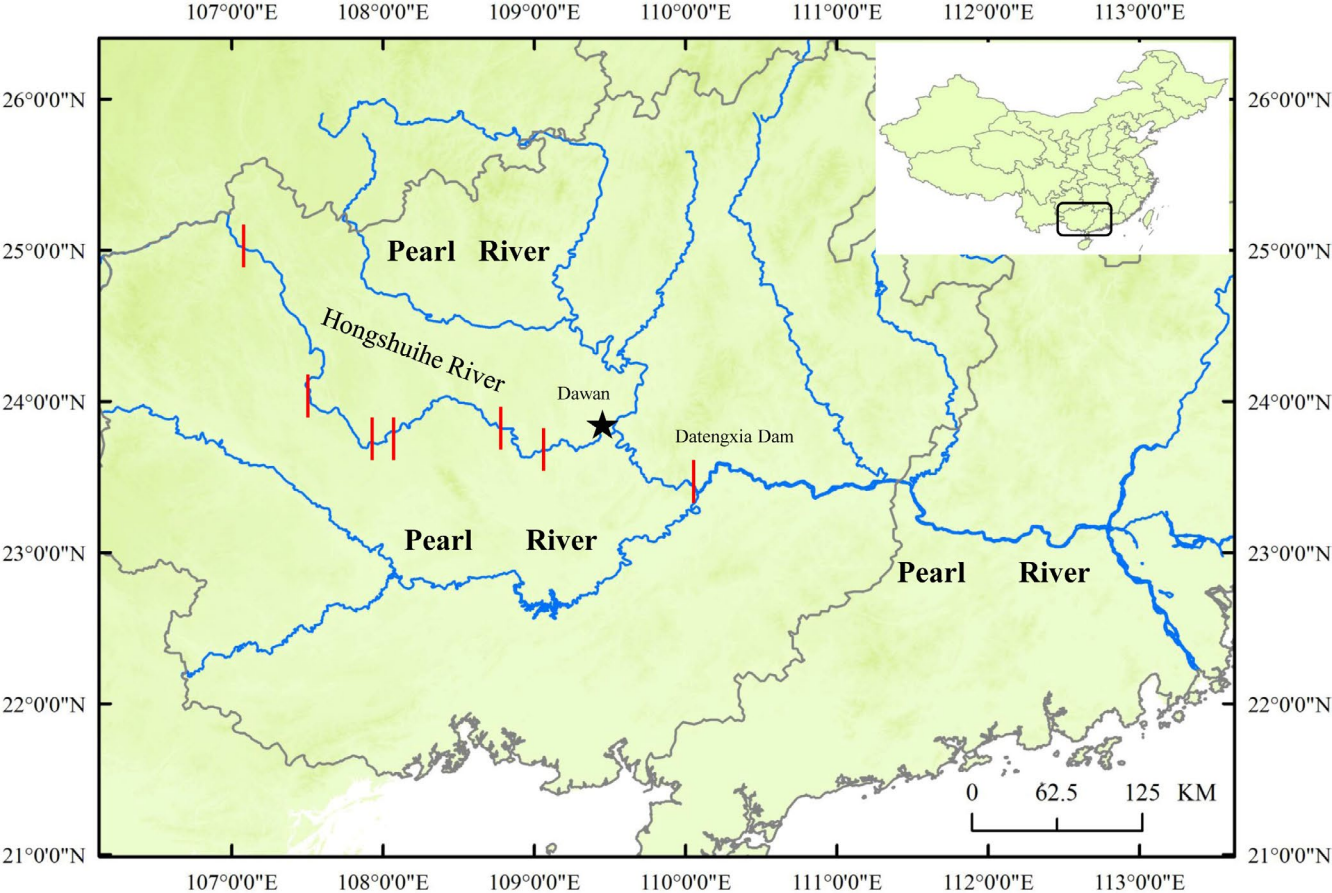
Map of the localities where eggs were sampled in this study (star). Red lines indicate the dams constructed in the Hongshuihe River and adjacent sections

The Hongshuihe River, an upper mainstem of the Pearl River, harbors rich fish diversity and many endemic fish species (Zheng, 1989; Zhou & Zhang, 2005). However, ongoing anthropogenic activities, such as hydraulic engineering, have severely affected the fish diversity in the Hongshuihe River (Dudgeon, 2011; Wang et al., 2015, 2019). For example, Wang et al. (2015) found obvious reductions in fish species richness in the mainstem of the Hongshuihe River and showed that small species dominated fish harvests. To date, six hydropower cascades (Figure 1) are located in the mainstem of the Hongshuihe River, and the largest dam in the Pearl River (Datengxia Dam) was constructed downstream of the Hongshuihe River in 2020 (Jian et al., 2010; Wang et al., 2015). These installations are likely to heavily pressure the fish populations in the Hongshuihe River. Hence, protecting the fish resources in the Hongshuihe River is a necessary task.

It is important to characterize the community compositions of fish resources during early development and spawning in particular regions in order to develop effective monitoring and conservation policies. In this study, we used DNA barcoding to determine the species composition of fish eggs in the lower Hongshuihe River (LHR) and to infer the corresponding reproductive periods of the identified species. Given that previous study found that most species in the Pearl River spawn during May and September (Shuai et al., 2016), so sampling was performed between May and September. Our main aims were: (a) to reveal the community composition of the egg pool, and (b) to predict the spawning periods of the identified species.

## 2 METHODS AND MATERIALS

### 2.1 Collection of fish eggs

Between May and September 2020, we collected fish eggs in Dawan town section of the LHR (Laibin City, Guangxi Province, China; 23.85457°N, 109.42775°E) (Figure 1) three times daily: at 6:00–8:00, 13:00–15:00, and 18:00–20:00. Empirical observations of this river section between 2011 and 2019 indicated that early‐stage eggs were most common fish resources. Two parallel customized nets (total length 2 m, rectangular iron opening/mouth 1.0 × 1.5 m, mesh size 0.5 mm; each attached to filtrate collection bucket 0.8 × 0.4 × 0.4 m) were anchored 10 m from the shoreline. Fish eggs in one net were preserved in 5% formalin, and eggs in the other net were stored in 95% ethanol. In total, the river was sampled 459 times; some of these samples did not yield any fish eggs.

### 2.2 Molecular experiment

Eggs preserved in 95% ethanol were used for the molecular analysis. Because it was impractical to amplify and sequence all of the collected eggs, we selected partial samples for molecular identification. Every week, we selected the sample with the most abundant fish eggs at each time slot for molecular identification, yielding 12 samples per month. Very few eggs were recovered in September; indeed, only one egg was caught in the third week. Among the 60 selected samples for molecular analysis, 15 did not include any fish eggs. Across all samples, 17 fish larvae were recovered. When selecting eggs from a given sample for molecular identification, we attempted to select eggs with different morphological features under the microscope. We examined the morphological features of 1,284 eggs and 17 larvae. Based on this tentative morphological assessment, we selected 641 eggs and 17 larvae for molecular identification (Table S1).

Total genomic DNA was extracted using the Axygen DNA Extraction Kit and checked using 1% agarose gel electrophoresis. A partial fragment of the 5'‐end of the mitochondrial COI gene (~648 bp) was amplified using the universal fish primers FishF1 and FishR1 (Ward et al., 2005). The PCR cycling conditions were as follows: initial denaturation at 95°C for 5 min; 30 cycles of 94°C for 30 s, 54°C for 30 s, and 72°C for 1 min; and a final extension at 72°C for 10 min. The PCR products were sequenced bidirectionally on an ABI 3730XL DNA system (Perkin‐Elmer Applied Biosystems, Foster City, CA, USA), following the manufacturer's protocols.

### 2.3 Data analyses

The tracer files and assembled sequences for the fish eggs and larvae were quality checked using the Lasergene package (DNASTAR, Inc., Madison, WI, USA). The high‐quality sequences were aligned using MUSCLE (Edgar, 2004) and edited by eye. A sequential approach was used to delimit species in the selected egg and larvae. First, the egg or larval sequences performed further identification in Barcode of Life Data System (BOLD). We retained the reference sequences of the best and second‐best interspecific matches in BOLD and documented the percentages of sequence matches. Unknown sequences that were more than 99% similar to the best sequence match and were less than 99% similar to second‐best match were unambiguously tagged with the species name of the best match (Hebert et al., 2004; Hubert et al., 2015). If the unknown sequence was more than 99% similar to both the best match and the second‐best match, and the two matches corresponded to the same genus and/or species, the unknown sequence was identified to genus level. Additionally, if the top 100 matches of the unknown sequence target to the same species, the unknown sequence was delimited to the targeted species. Second, if egg and larval sequences were not identified into species level in BOLD database, these sequences were compared to the GenBank nucleotide database using the Basic Local Alignment Search Tool (BLAST; https://blast.ncbi.nlm.nih.gov/Blast.cgi). If the top five BLAST results represented the same species, and if sequence identity was more than 99%, the unknown sequence was considered accurately identified. If sequence identity between the unknown sequence and the top BLAST results was greater than 95%, the unknown sequence was identified to genus level. Sequences with less than 95% identity to any sequence in the GenBank database were considered unidentified. Lastly, a neighbor‐joining (NJ) tree was constructed in MEGA version 6 (Tamura et al., 2013) with 1,000 bootstrap replicates using the Kimura‐2 parameter model (Kimura, 1980) for illustrating the identification accuracy of the two abovementioned approaches.

## 3 RESULTS

### 3.1 Sequence information

We obtained 397 and 17 high‐quality sequences from 641 eggs and 17 larvae, respectively (Table S1). Due to poor sample preservation, many of the eggs did not yield PCR products or high‐quality sequences (*n* = 244). After sequence alignment and the removal of noisy sites, we obtained 569‐bp DNA barcodes for 414 specimens. All egg sequences that were identified to species level were deposited in the GenBank database (Table S1).

### 3.2 Molecular identification of fish eggs

Utilizing a 1% divergence threshold to represent species boundaries in BOLD database, 392 egg sequences were successfully assigned to 10 species, and nine larval sequences were identified to four species (Table S2). We then used BLAST to search the remaining five egg and eight larval sequences, which could not be diagnosed into species level using the BOLD database, against the GenBank database. Four larval sequences were targeted to two species (*Siniperca scherzeri* and *Siniperca roulei*) with more than 99% similarity in BOLD, while BLAST results suggested that the four larval sequences corresponded to *Siniperca scherzeri* with 100% identity in the top 5 matches (Table S3). Considering that *Siniperca roulei* was a rare species and was not discovered in recent field surveys (Wang et al., 2019), we argued that the four larval sequences could be assigned into *Siniperca scherzeri*. Furthermore, BLAST results found that two larval sequences fell into the genus *Rhinogobius*, and two eggs corresponded to the genus *Xenocypris*. The final three egg and one larval sequences could not be identified to genus or species based on BOLD and GenBank databases (Tables S3 and S4). In total, we successfully assigned 392 eggs (~98.7%) to 10 species and two eggs to one genus. We also successfully assigned 13 larvae to four species and two larvae to one genus. Overall, the identified eggs and larvae fell into 15 genera and five families. Among the 14 species identified (Table 1), the family Cyprinidae (10 species) was most abundant, accounting for approximately 71% of all species identified.

**TABLE 1:** The community composition, occurrence frequency, and occurrence times of taxa identified in fish egg pool between May and September using DNA barcoding

Family	Species	May	June	July	August	September
Cyprinidae	*Pseudohemiculter dispar*	+	+	+	+	
Cyprinidae	*Squaliobarbus curriculus*		+		+	
Cyprinidae	*Ctenopharyngodon idella*		+			
Cyprinidae	*Xenocypris* spp		+			
Cyprinidae	*Zacco platypus*				+	
Cyprinidae	*Pseudolaubuca sinensis*	+				
Cyprinidae	*Squalidus argentatus*	+	+	+	+	
Cyprinidae	*Gobiobotia meridionalis*	+	+	+	+	
Cyprinidae	*Onychostoma gerlachi*	+	+		+	
Cyprinidae	*Garra orientalis*		+			
Cyprinidae	*Sinogastromyzon wui*	+				
Botiidae	*Sinibotia pulchra*		+			
Botiidae	*Sinibotia robusta*			+	+	+
Mastacembelidae	*Mastacembelus armatus*	+	+	+	+	
Serranidae	*Siniperca scherzeri*				+	
Gobiidae	*Rhinogobius* spp1		+	+	+	
Gobiidae	*Rhinogobius* spp2			+		
	Unknown species 1			+		
	Unknown species 2			+		
	Total	7	11	9	10	1

Note

Plus sign (+) indicates occurrence of identified species in each month.

**FIGURE 2:**
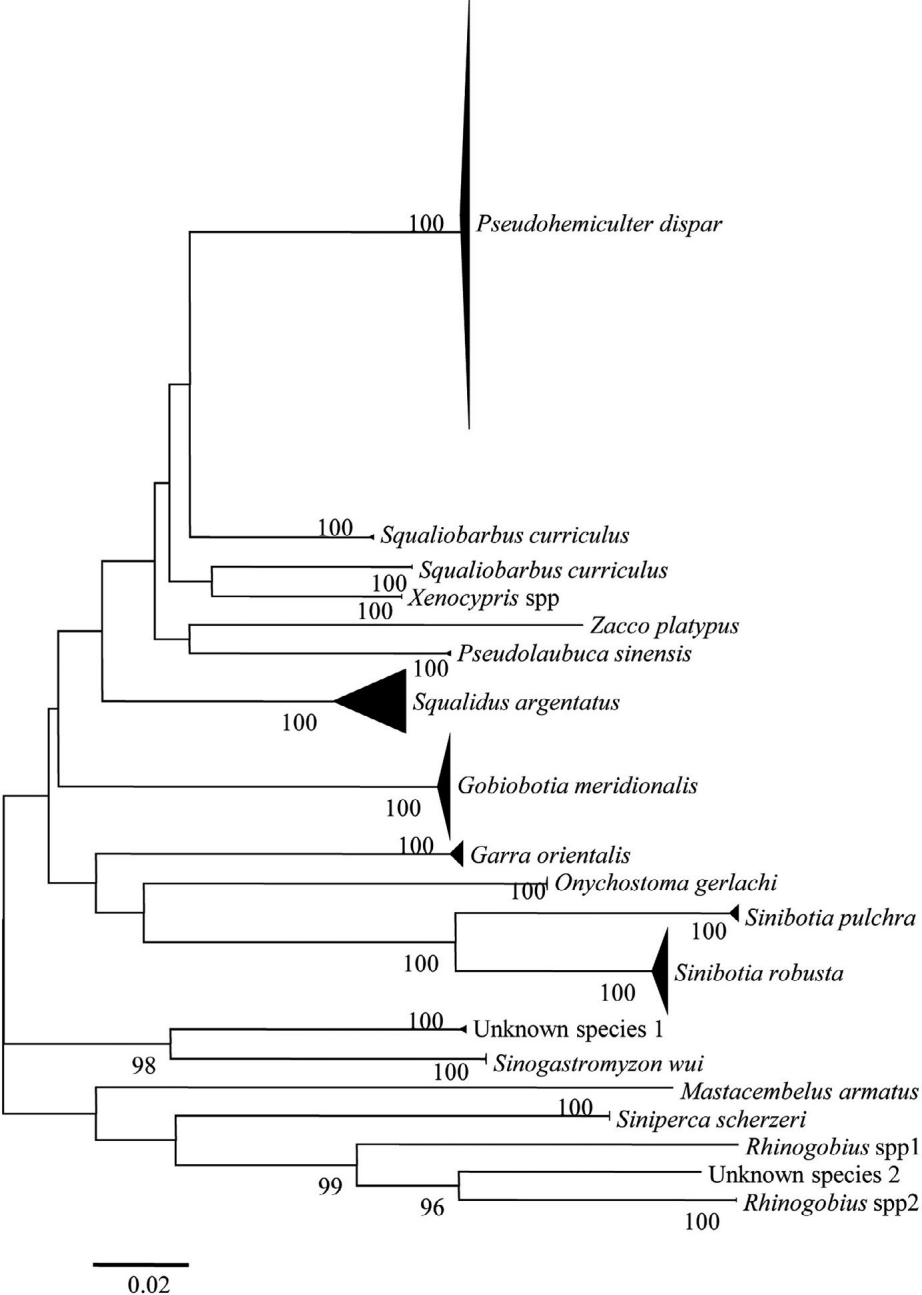
Neighbor‐joining tree of 397 eggs and 17 larvae. Bootstrap values are given at the nodes

The NJ tree suggested that diagnosed species based on BOLD and GenBank databases generated 19 independent lineages (Figure 2), suggesting that at least 19 distinct species were found in the all samples and the eggs and/or larvae were accurately identified in this study. Each month, we collected 1–11 species (Table 1), with species numbers lowest in September (only one species) and highest in June (11 species). Five of the 14 species identified (*Pseudohemiculter dispar*, *Squalidus argentatus*, *Gobiobotia meridionalis*, *Onychostoma gerlachi,* and *Sinibotia robusta*) had long spawning periods (May to August), indicating that these species may dominate the studied river section (Table 1). Two additional species, *Sinibotia pulchra* and *Siniperca scherzeri,* had spawning periods that lasted at least three months (Table 1).

## 4 DISCUSSION

### 4.1 High failure rate of fish egg amplification

In this study, the failure rate of DNA extraction or amplification was high (~38%). This low success rate was most likely due to poor storage conditions (low ethanol concentrations for long periods) prior to molecular analysis. Although the fish eggs were preserved in 95% ethanol, water was inadvertently added to the storage boxes with the eggs, lowering the ethanol concentration. In addition, eggs were preserved in these boxes for more than 6 months before DNA extraction, and the ethanol was not replaced during this time. A previous study suggested that DNA degradation accelerates after 6 months of storage in ethanol (Michaud & Foran, 2011). Thus, the low ethanol concentration over the long‐term storage period negatively affected DNA stability and led to DNA degradation (Michaud & Foran, 2011). In future studies, we will change ethanol for a couple of times shortly after the collection to get better preserved samples and will process the eggs more quickly (i.e., within 6 months) to improve DNA quality and yield.

### 4.2 Community composition

Across all samples, we successfully identified 14 species in 13 genera and four families. Ten of the 14 identified species were cyprinids (~71%), suggesting that the family Cyprinidae is a dominant fish group in the LHR. A similar proportion of cyprinids was recovered via field sampling in the mainstem of the Hongshuihe River in 2009 and 2018 (Wang et al., 2019). In this previous study, two field surveys identified 49 and 39 cyprinids in the mainstem, accounting for ca. 57% and 63%, respectively, of all species identified (89 species in 2009 and 62 species in 2018) (Wang et al., 2019). *Pseudohemiculter dispar*, *Squalidus argentatus,* and *Sinibotia robusta* occurred frequently in our samples, suggesting that these species are unambiguously dominant in the LHR, which was consistent with the findings of the previous study in 2009 and 2018 (Wang et al., 2019). A previous study focused on the early stages of fish development in the LHR also suggested that these three species were dominant in this river section (Gao, 2018). Furthermore, *Gobiobotia meridionalis* was frequently occurred in the egg pool, suggesting this species might be relatively abundant in the Hongshuihe River. However, this finding was not in line with the results from previous field surveys (Wang et al., 2015, 2019), which did not uncover *Gobiobotia meridionalis* in this river. *Gobiobotia meridionalis* is not economically valuable and may be ignored by fishermen, so this species might be difficult to be seen during field surveys. This example highlights the advantage of using our approach to identify species in the egg pool, as this approach may capture species that occur in the region only infrequently as adults. Thus, our approach provides another method of assessing species stocks.

With the exception of the two migratory species identified in this study (*Squaliobarbus curriculus* and *Ctenopharyngodon idella*), the remaining 12 species and two *Rhinogobius* species that cannot be assigned to species level are small and/or benthic‐resident species (Zheng, 1989; Zhou & Zhang, 2005). This result was consistent with the findings of Wang et al. (2015), who found that middle‐ and/or small‐sized species dominated the species community in field surveys. The low numbers of migratory species and high numbers of small species observed in our study might be partially due to the development of hydropower cascades in this river section and adjacent regions. In addition to the Datengxia Dam downstream of the Hongshuihe River, six hydropower cascades have been constructed in the mainstem of the Hongshuihe River (Jian et al., 2010; Wang et al., 2015). These cascades may block the migration and reproductive behavior of some dispersal species, which decreases the likelihood that these species will be detected by field surveys during early development or as adults. Small and/or benthic species are less influenced by cascades because these species do not migrate long distances. These factors suggested that our study provides a novel perspective on the fish community in the studied region. Given that this study chose a partial of samples for molecular identification and high failure rate of amplification, species count of the egg pool in the LHR might be underestimated and the stochastic error would be occurred in our inferences involving in fish community.

### 4.3 Spawning periods of fish species in the LHR

Fish spawning periods can reflect spawning activity, and a better understanding of these periods can support long‐term fish monitoring efforts during spawning (Carassou & Ponton, 2007; Jakobsen et al., 2016; Pritt et al., 2015). Given that spawning times tend to be both species‐ and region‐specific (Cao et al., 2007; Jakobsen et al., 2016), it is important to determine the spawning periods for as many species as possible in a given region. To date, only a single study has assessed the early life stages of fish resources in the LHR (Gao, 2018). However, this previous study identified a majority of the eggs and larvae to the species level based on morphological characters, and thus, many species were not detected (Gao, 2018). Environmental variabilities among river sections may cause identical species to spawn at different times in different locations (Jakobsen et al., 2016). Thus, the spawning periods predicted in this study might only apply to fish species in the LHR.

We found that the number of species identified varied from 7 to 11 between May and August and that spawning period differed among species, implying that the environmental conditions required for reproductive behavior may be species‐specific. Five species (*Pseudohemiculter dispar*, *Squalidus argentatus*, *Gobiobotia meridionalis*, *Onychostoma gerlachi,* and *Sinibotia robusta*) spawned continuously for long periods (at least four months of the year). Of these, the four small, benthic species (*Pseudohemiculter dispar*, *Squalidus argentatus*, *Gobiobotia meridionalis*, and *Sinibotia robusta*) likely had longer spawning periods due to their shorter time to sexual maturity, sedentary lifestyle, and/or high adaptive capacity (Zhou & Zhang, 2005). *Onychostoma gerlachi*, a species that prefers to reside in middle and lower water layer and is deemed a poor disperser, may spawn for longer periods due to its sedentary lifestyle (Zhou & Zhang, 2005). Two species in the genus *Sinibotia*, *Sinibotia pulchra* and *Siniperca scherzeri*, spawned for at least three months. A previous study of reproductive periods in the Pearl River indicated that *Siniperca kneri* spawned between April and August (Wang et al., 2006), suggesting that we may have underestimated the spawning periods of these species. Indeed, because we did not sample throughout the year, we may have underestimated the reproductive periods of several fish in the LHR.

## 5 CONCLUSIONS

After high‐density egg sampling during the peak reproductive season, we used DNA barcoding techniques to explore the species composition of the egg pool in the LHR and to infer the spawning periods of identified species. We successfully classified 392 eggs and 13 larvae into 14 species. This represented a substantial improvement in identification success compared with traditional identification methods, which are based on morphological characteristics of the eggs or larvae. However, it was impossible to use traditional DNA barcoding to analyze each egg collected because the sample size was excessively large. DNA metabarcoding, which can obtain hundreds of thousands of barcode sequences from mixed samples (Kimmerling et al., 2018; Klymus et al., 2017), represents an effective method with which to delimit species in large samples of fish eggs and/or larvae. Thus, DNA metabarcoding may provide a more detailed and comprehensive overview of the community composition of egg and/or larval pools.

## CONFLICT OF INTEREST

The authors declare no competing financial interest.

## AUTHOR CONTRIBUTIONS

**Weitao Chen:** Conceptualization (lead); Conceptualization (lead); Methodology (lead); Software (lead); Software (lead); Writing‐original draft (lead); Writing‐original draft (lead). **Shuli Zhu:** Investigation (equal); Resources (equal); Software (equal). **Jiping Yang:** Investigation (equal); Resources (equal). **Xinhui Li:** Conceptualization (equal); Funding acquisition (equal). **Yuefei Li:** Conceptualization (equal); Investigation (lead); Resources (equal). **Jie Li:** Conceptualization (equal); Supervision (equal); Visualization (equal).

## Supporting information

Table S1

Table S2

Table S3

Table S4

## Data Availability

DNA sequences have been deposited in GenBank under Accession numbers MZ148846‐MZ149250. Details regarding individual samples are available in Table S1.
