# Supplementary material for: DNA barcoding reveals the temporal community composition of drifting fish eggs in the lower Hongshui River, China
Source: Ecol Evol. 2021 Jul 22;11(16):11507–14. doi: 10.1002/ece3.7943 (PMC8366882; doi:10.1002/ece3.7943)
Supplement: Supplementary file 1 — Table S1 [file ECE3-11-11507-s003.docx]

| Sample date | Sample time | Sample ID | Species | Genbank Accession |
| --- | --- | --- | --- | --- |
| 2020/5/2 | morning | DW1 | *Pseudohemiculter dispar* | MZ148918 |
| 2020/5/2 | morning | DW2 | *Pseudohemiculter dispar* | MZ148998 |
| 2020/5/2 | morning | DW3 | *Pseudohemiculter dispar* | MZ149033 |
| 2020/5/2 | morning | DW4 | *Pseudohemiculter dispar* | MZ149034 |
| 2020/5/2 | morning | DW5 | *Pseudohemiculter dispar* | MZ149036 |
| 2020/5/2 | morning | DW7 | *Pseudohemiculter dispar* | MZ149067 |
| 2020/5/2 | morning | DW8 | *Pseudohemiculter dispar* | MZ149097 |
| 2020/5/2 | morning | DW9 | *Pseudohemiculter dispar* | MZ149129 |
| 2020/5/2 | morning | DW10 | *Pseudohemiculter dispar* | MZ148919 |
| 2020/5/2 | morning | DW11 | *Pseudohemiculter dispar* | MZ148920 |
| 2020/5/2 | morning | DW12 | *Pseudohemiculter dispar* | MZ148951 |
| 2020/5/2 | morning | DW41 | *Pseudohemiculter dispar* | MZ149035 |
| 2020/5/10 | morning | DW0091 | *Pseudohemiculter dispar* | MZ148911 |
| 2020/5/10 | morning | DW0094 | *Gobiobotia meridionalis* | MZ148859 |
| 2020/5/10 | morning | DW0097 | *Pseudohemiculter dispar* | MZ148912 |
| 2020/5/10 | morning | DW0099 | *Pseudohemiculter dispar* | MZ148913 |
| 2020/5/10 | morning | DW0101 | *Pseudohemiculter dispar* | MZ148914 |
| 2020/5/10 | morning | DW0103 | *Pseudohemiculter dispar* | MZ148915 |
| 2020/5/10 | morning | DW0104 | *Sinibotia robusta* | MZ149174 |
| 2020/5/10 | morning | DW0105 | *Pseudohemiculter dispar* | MZ148916 |
| 2020/5/10 | morning | DW0106 | *Sinibotia robusta* | MZ149175 |
| 2020/5/10 | morning | DW111 | *Gobiobotia meridionalis* | MZ148860 |
| 2020/5/10 | morning | DW113 | *Sinibotia robusta* | MZ149177 |
| 2020/5/10 | morning | DW114 | *Sinogastromyzon wui* | MZ149215 |
| 2020/5/10 | morning | DW115 | *Pseudohemiculter dispar* | MZ148927 |
| 2020/5/10 | morning | DW116 | *Pseudohemiculter dispar* | MZ148936 |
| 2020/5/10 | morning | DW117 | *Pseudohemiculter dispar* | MZ148943 |
| 2020/5/10 | morning | DW119 | *Sinibotia robusta* | MZ149178 |
| 2020/5/10 | morning | DW120 | *Sinibotia robusta* | MZ149179 |
| 2020/5/10 | morning | DW122 | *Sinogastromyzon wui* | MZ149216 |
| 2020/5/10 | morning | DW125 | *Pseudohemiculter dispar* | MZ148958 |
| 2020/5/10 | morning | DW127 | *Pseudohemiculter dispar* | MZ148959 |
| 2020/5/10 | morning | DW128 | *Pseudohemiculter dispar* | MZ148960 |
| 2020/5/10 | morning | DW129 | *Pseudohemiculter dispar* | MZ148961 |
| 2020/5/10 | morning | DW130 | *Pseudohemiculter dispar* | MZ148962 |
| 2020/5/10 | morning | DW131 | *Pseudohemiculter dispar* | MZ148963 |
| 2020/5/10 | morning | DW132 | *Pseudohemiculter dispar* | MZ148964 |
| 2020/5/10 | morning | DW0134 | *Pseudohemiculter dispar* | MZ148917 |
| 2020/5/10 | morning | DW135 | *Pseudohemiculter dispar* | MZ148965 |
| 2020/5/10 | morning | DW136 | *Pseudohemiculter dispar* | MZ148966 |
| 2020/5/10 | morning | DW137 | *Gobiobotia meridionalis* | MZ148863 |
| 2020/5/10 | morning | DW138 | *Pseudohemiculter dispar* | MZ148967 |
| 2020/5/10 | morning | DW139 | *Sinogastromyzon wui* | MZ149217 |
| 2020/5/10 | morning | DW140 | *Pseudohemiculter dispar* | MZ148968 |
| 2020/5/10 | morning | DW141 | *Pseudohemiculter dispar* | MZ148969 |
| 2020/5/10 | morning | DW142 | *Pseudohemiculter dispar* | MZ148970 |
| 2020/5/10 | morning | DW143 | *Sinogastromyzon wui* | MZ149218 |
| 2020/5/10 | morning | DW144 | *Gobiobotia meridionalis* | MZ148864 |
| 2020/5/10 | morning | DW145 | *Pseudohemiculter dispar* | MZ148971 |
| 2020/5/10 | morning | DW147 | *Pseudohemiculter dispar* | MZ148972 |
| 2020/5/10 | morning | DW148 | *Sinibotia robusta* | MZ149194 |
| 2020/5/10 | morning | DW149 | *Pseudohemiculter dispar* | MZ148973 |
| 2020/5/10 | morning | DW151 | *Pseudohemiculter dispar* | MZ148974 |
| 2020/5/10 | morning | DW152 | *Gobiobotia meridionalis* | MZ148865 |
| 2020/5/10 | morning | DW153 | *Sinibotia robusta* | MZ149195 |
| 2020/5/10 | morning | DW154 | *Gobiobotia meridionalis* | MZ148866 |
| 2020/5/10 | morning | DW155 | *Pseudohemiculter dispar* | MZ148975 |
| 2020/5/10 | morning | DW157 | *Pseudohemiculter dispar* | MZ148976 |
| 2020/5/10 | morning | DW158 | *Pseudohemiculter dispar* | MZ148977 |
| 2020/5/10 | morning | DW159 | *Pseudohemiculter dispar* | MZ148978 |
| 2020/5/10 | morning | DW162 | *Gobiobotia meridionalis* | MZ148867 |
| 2020/5/10 | morning | DW166 | *Pseudohemiculter dispar* | MZ148979 |
| 2020/5/10 | morning | DW168 | *Sinogastromyzon wui* | MZ149219 |
| 2020/5/10 | morning | DW170 | *Pseudohemiculter dispar* | MZ148980 |
| 2020/5/10 | morning | DW171 | *Pseudohemiculter dispar* | MZ148981 |
| 2020/5/10 | morning | DW172 | *Pseudohemiculter dispar* | MZ148982 |
| 2020/5/10 | morning | DW173 | *Pseudohemiculter dispar* | MZ148983 |
| 2020/5/10 | morning | DW176 | *Pseudohemiculter dispar* | MZ148984 |
| 2020/5/10 | morning | DW178 | *Pseudohemiculter dispar* | MZ148985 |
| 2020/5/10 | morning | DW180 | *Pseudohemiculter dispar* | MZ148986 |
| 2020/5/10 | morning | DW186 | *Pseudohemiculter dispar* | MZ148987 |
| 2020/5/10 | morning | DW187 | *Pseudohemiculter dispar* | MZ148988 |
| 2020/5/10 | morning | DW188 | *Pseudohemiculter dispar* | MZ148989 |
| 2020/5/10 | morning | DW189 | *Sinibotia robusta* | MZ149196 |
| 2020/5/10 | morning | DW191 | *Pseudohemiculter dispar* | MZ148990 |
| 2020/5/10 | morning | DW193 | *Pseudohemiculter dispar* | MZ148991 |
| 2020/5/10 | morning | DW194 | *Pseudohemiculter dispar* | MZ148992 |
| 2020/5/10 | morning | DW195 | *Pseudohemiculter dispar* | MZ148993 |
| 2020/5/10 | morning | DW196 | *Pseudohemiculter dispar* | MZ148994 |
| 2020/5/10 | morning | DW197 | *Pseudohemiculter dispar* | MZ148995 |
| 2020/5/10 | morning | DW198 | *Pseudohemiculter dispar* | MZ148996 |
| 2020/5/10 | morning | DW199 | *Pseudohemiculter dispar* | MZ148997 |
| 2020/5/10 | morning | DW200 | *Pseudohemiculter dispar* | MZ148999 |
| 2020/5/10 | morning | DW201 | *Pseudohemiculter dispar* | MZ149000 |
| 2020/5/10 | morning | DW202 | *Pseudohemiculter dispar* | MZ149001 |
| 2020/5/10 | morning | DW203 | *Pseudohemiculter dispar* | MZ149002 |
| 2020/5/10 | morning | DW206 | *Pseudohemiculter dispar* | MZ149003 |
| 2020/5/10 | morning | DW207 | *Pseudohemiculter dispar* | MZ149004 |
| 2020/5/10 | morning | DW208 | *Pseudohemiculter dispar* | MZ149005 |
| 2020/5/10 | morning | DW209 | *Pseudohemiculter dispar* | MZ149006 |
| 2020/5/10 | morning | DW210 | *Pseudohemiculter dispar* | MZ149007 |
| 2020/5/10 | morning | DW211 | *Pseudohemiculter dispar* | MZ149008 |
| 2020/5/10 | morning | DW212 | *Pseudohemiculter dispar* | MZ149009 |
| 2020/5/10 | morning | DW213 | *Pseudolaubuca sinensis* | MZ149165 |
| 2020/5/10 | morning | DW214 | *Pseudohemiculter dispar* | MZ149010 |
| 2020/5/10 | morning | DW215 | *Pseudohemiculter dispar* | MZ149011 |
| 2020/5/10 | morning | DW216 | *Pseudohemiculter dispar* | MZ149012 |
| 2020/5/10 | morning | DW217 | *Pseudohemiculter dispar* | MZ149013 |
| 2020/5/10 | morning | DW218 | *Pseudohemiculter dispar* | MZ149014 |
| 2020/5/10 | morning | DW219 | *Pseudohemiculter dispar* | MZ149015 |
| 2020/5/10 | morning | DW220 | *Pseudohemiculter dispar* | MZ149016 |
| 2020/5/10 | morning | DW221 | *Pseudohemiculter dispar* | MZ149017 |
| 2020/5/10 | morning | DW223 | *Pseudohemiculter dispar* | MZ149018 |
| 2020/5/10 | morning | DW224 | *Pseudohemiculter dispar* | MZ149019 |
| 2020/5/10 | morning | DW225 | *Pseudohemiculter dispar* | MZ149020 |
| 2020/5/10 | morning | DW226 | *Pseudohemiculter dispar* | MZ149021 |
| 2020/5/10 | morning | DW227 | *Pseudohemiculter dispar* | MZ149022 |
| 2020/5/10 | morning | DW228 | *Pseudohemiculter dispar* | MZ149023 |
| 2020/5/10 | morning | DW229 | *Pseudohemiculter dispar* | MZ149024 |
| 2020/5/10 | morning | DW230 | *Pseudohemiculter dispar* | MZ149025 |
| 2020/5/10 | morning | DW231 | *Pseudolaubuca sinensis* | MZ149166 |
| 2020/5/10 | morning | DW234 | *Pseudohemiculter dispar* | MZ149026 |
| 2020/5/10 | morning | DW237 | *Pseudohemiculter dispar* | MZ149027 |
| 2020/5/10 | morning | DW238 | *Pseudohemiculter dispar* | MZ149028 |
| 2020/5/10 | morning | DW239 | *Pseudohemiculter dispar* | MZ149029 |
| 2020/5/10 | morning | DW242 | *Pseudohemiculter dispar* | MZ149030 |
| 2020/5/10 | morning | DW243 | *Pseudohemiculter dispar* | MZ149031 |
| 2020/5/10 | morning | DW244 | *Pseudohemiculter dispar* | MZ149032 |
| 2020/5/10 | morning | DW300 | *Sinibotia robusta* | MZ149197 |
| 2020/5/10 | noon | DW326 | *Sinibotia robusta* | MZ149198 |
| 2020/5/26 | morning | DW414 | *Squalidus argentatus* | MZ149236 |
| 2020/5/26 | morning | DW439 | *Gobiobotia meridionalis* | MZ148868 |
| 2020/5/26 | morning | DW440 | *Squalidus argentatus* | MZ149237 |
| 2020/5/26 | morning | DW442 | *Gobiobotia meridionalis* | MZ148869 |
| 2020/5/26 | morning | DW443 | *Gobiobotia meridionalis* | MZ148870 |
| 2020/5/26 | morning | DW448 | *Gobiobotia meridionalis* | MZ148871 |
| 2020/5/26 | morning | DW450 | *Gobiobotia meridionalis* | MZ148872 |
| 2020/5/26 | morning | DW452 | *Gobiobotia meridionalis* | MZ148873 |
| 2020/5/26 | morning | DW455 | *Gobiobotia meridionalis* | MZ148874 |
| 2020/5/26 | noon | DW459 | *Gobiobotia meridionalis* | MZ148875 |
| 2020/5/26 | noon | DW460 | *Gobiobotia meridionalis* | MZ148876 |
| 2020/5/26 | noon | DW461 | *Gobiobotia meridionalis* | MZ148877 |
| 2020/5/26 | noon | DW469 | *Gobiobotia meridionalis* | MZ148878 |
| 2020/5/26 | noon | DW471 | *Gobiobotia meridionalis* | MZ148879 |
| 2020/5/26 | noon | DW472 | *Gobiobotia meridionalis* | MZ148880 |
| 2020/5/26 | noon | DW474 | *Gobiobotia meridionalis* | MZ148881 |
| 2020/5/26 | noon | DW475 | *Gobiobotia meridionalis* | MZ148882 |
| 2020/5/26 | noon | DW476 | *Gobiobotia meridionalis* | MZ148883 |
| 2020/5/26 | noon | DW484 | *Gobiobotia meridionalis* | MZ148884 |
| 2020/5/26 | noon | DW486 | *Gobiobotia meridionalis* | MZ148885 |
| 2020/5/26 | noon | DW487 | *Gobiobotia meridionalis* | MZ148886 |
| 2020/5/26 | noon | DW488 | *Gobiobotia meridionalis* | MZ148887 |
| 2020/5/26 | night | DW494 | *Gobiobotia meridionalis* | MZ148888 |
| 2020/5/26 | night | DW495 | *Gobiobotia meridionalis* | MZ148889 |
| 2020/5/26 | night | DW498 | *Gobiobotia meridionalis* | MZ148890 |
| 2020/5/26 | night | DW500 | *Gobiobotia meridionalis* | MZ148891 |
| 2020/5/26 | night | DW504 | *Gobiobotia meridionalis* | MZ148892 |
| 2020/5/26 | night | DW512 | *Gobiobotia meridionalis* | MZ148893 |
| 2020/6/2 | noon | DW533 | *Gobiobotia meridionalis* | MZ148894 |
| 2020/6/2 | night | DW539 | *Gobiobotia meridionalis* | MZ148895 |
| 2020/6/18 | night | DW608 | *Pseudohemiculter dispar* | MZ149037 |
| 2020/6/26 | morning | DW615 | *Sinibotia robusta* | MZ149199 |
| 2020/6/26 | morning | DW616 | Unknown species 1 | / |
| 2020/6/26 | morning | DW617 | *Sinibotia robusta* | MZ149200 |
| 2020/6/26 | morning | DW618 | *Squaliobarbus curriculus* | MZ149249 |
| 2020/6/26 | morning | DW619 | *Sinibotia robusta* | MZ149201 |
| 2020/6/26 | morning | DW620 | *Xenocypris* sp | / |
| 2020/6/26 | morning | DW621 | *Garra orientalis* | MZ148848 |
| 2020/6/26 | morning | DW622 | *Sinibotia robusta* | MZ149202 |
| 2020/6/26 | morning | DW623 | *Sinibotia robusta* | MZ149203 |
| 2020/6/26 | morning | DW624 | *Sinibotia robusta* | MZ149204 |
| 2020/6/26 | morning | DW625 | *Xenocypris* sp | / |
| 2020/6/26 | morning | DW626 | *Sinibotia robusta* | MZ149205 |
| 2020/6/26 | morning | DW627 | *Sinibotia robusta* | MZ149206 |
| 2020/6/26 | morning | DW628 | *Garra orientalis* | MZ148849 |
| 2020/6/26 | morning | DW629 | *Sinibotia robusta* | MZ149207 |
| 2020/6/26 | morning | DW630 | *Garra orientalis* | MZ148850 |
| 2020/6/26 | morning | DW631 | *Sinibotia robusta* | MZ149208 |
| 2020/6/26 | morning | DW632 | *Sinibotia robusta* | MZ149209 |
| 2020/6/26 | noon | DW633 | *Ctenopharyngodon idella* | MZ148846 |
| 2020/6/26 | noon | DW634 | *Garra orientalis* | MZ148851 |
| 2020/6/26 | noon | DW635 | *Garra orientalis* | MZ148852 |
| 2020/6/26 | noon | DW636 | *Ctenopharyngodon idella* | MZ148847 |
| 2020/6/26 | noon | DW638 | *Garra orientalis* | MZ148853 |
| 2020/6/26 | noon | DW639 | *Garra orientalis* | MZ148854 |
| 2020/6/26 | noon | DW640 | *Garra orientalis* | MZ148855 |
| 2020/6/26 | noon | DW645 | Unknown species 1 | / |
| 2020/6/26 | noon | DW646 | *Garra orientalis* | MZ148856 |
| 2020/6/26 | noon | DW647 | *Sinibotia robusta* | MZ149210 |
| 2020/6/26 | noon | DW653 | *Garra orientalis* | MZ148857 |
| 2020/6/26 | noon | DW654 | *Garra orientalis* | MZ148858 |
| 2020/6/26 | noon | DW655 | Unknown species 1 | / |
| 2020/6/26 | night | DW661 | *Squalidus argentatus* | MZ149238 |
| 2020/7/2 | morning | DW663 | *Pseudohemiculter dispar* | MZ149038 |
| 2020/7/2 | morning | DW664 | *Pseudohemiculter dispar* | MZ149039 |
| 2020/7/2 | morning | DW665 | *Pseudohemiculter dispar* | MZ149040 |
| 2020/7/2 | morning | DW666 | *Pseudohemiculter dispar* | MZ149041 |
| 2020/7/2 | morning | DW667 | *Pseudohemiculter dispar* | MZ149042 |
| 2020/7/2 | morning | DW669 | *Pseudohemiculter dispar* | MZ149043 |
| 2020/7/2 | morning | DW670 | *Pseudohemiculter dispar* | MZ149044 |
| 2020/7/2 | morning | DW671 | *Pseudohemiculter dispar* | MZ149045 |
| 2020/7/2 | morning | DW672 | *Pseudohemiculter dispar* | MZ149046 |
| 2020/7/2 | morning | DW673 | *Pseudohemiculter dispar* | MZ149047 |
| 2020/7/2 | morning | DW674 | *Pseudohemiculter dispar* | MZ149048 |
| 2020/7/2 | morning | DW675 | *Pseudohemiculter dispar* | MZ149049 |
| 2020/7/2 | morning | DW676 | *Pseudohemiculter dispar* | MZ149050 |
| 2020/7/2 | morning | DW677 | *Pseudohemiculter dispar* | MZ149051 |
| 2020/7/2 | morning | DW678 | *Pseudohemiculter dispar* | MZ149052 |
| 2020/7/2 | morning | DW679 | *Pseudohemiculter dispar* | MZ149053 |
| 2020/7/2 | morning | DW680 | *Pseudohemiculter dispar* | MZ149054 |
| 2020/7/2 | morning | DW681 | *Pseudohemiculter dispar* | MZ149055 |
| 2020/7/2 | morning | DW683 | *Pseudohemiculter dispar* | MZ149056 |
| 2020/7/2 | morning | DW684 | *Pseudohemiculter dispar* | MZ149057 |
| 2020/7/2 | morning | DW685 | *Pseudohemiculter dispar* | MZ149058 |
| 2020/7/2 | morning | DW686 | *Pseudohemiculter dispar* | MZ149059 |
| 2020/7/2 | morning | DW687 | *Pseudohemiculter dispar* | MZ149060 |
| 2020/7/2 | morning | DW688 | *Pseudohemiculter dispar* | MZ149061 |
| 2020/7/2 | morning | DW690 | *Pseudohemiculter dispar* | MZ149062 |
| 2020/7/2 | morning | DW691 | *Pseudohemiculter dispar* | MZ149063 |
| 2020/7/2 | morning | DW692 | *Pseudohemiculter dispar* | MZ149064 |
| 2020/7/2 | morning | DW695 | *Pseudohemiculter dispar* | MZ149065 |
| 2020/7/2 | morning | DW696 | *Pseudohemiculter dispar* | MZ149066 |
| 2020/7/2 | morning | DW701 | *Sinibotia pulchra* | MZ149172 |
| 2020/7/2 | noon | DW733 | *Pseudohemiculter dispar* | MZ149068 |
| 2020/7/2 | noon | DW734 | *Pseudohemiculter dispar* | MZ149069 |
| 2020/7/2 | noon | DW735 | *Squalidus argentatus* | MZ149239 |
| 2020/7/2 | noon | DW737 | *Pseudohemiculter dispar* | MZ149070 |
| 2020/7/2 | noon | DW738 | *Pseudohemiculter dispar* | MZ149071 |
| 2020/7/2 | noon | DW739 | *Pseudohemiculter dispar* | MZ149072 |
| 2020/7/2 | noon | DW741 | *Pseudohemiculter dispar* | MZ149073 |
| 2020/7/2 | noon | DW742 | *Pseudohemiculter dispar* | MZ149074 |
| 2020/7/2 | noon | DW743 | *Pseudohemiculter dispar* | MZ149075 |
| 2020/7/2 | noon | DW744 | *Pseudohemiculter dispar* | MZ149076 |
| 2020/7/2 | noon | DW745 | *Pseudohemiculter dispar* | MZ149077 |
| 2020/7/2 | noon | DW746 | *Pseudohemiculter dispar* | MZ149078 |
| 2020/7/2 | noon | DW747 | *Pseudohemiculter dispar* | MZ149079 |
| 2020/7/2 | noon | DW748 | *Pseudohemiculter dispar* | MZ149080 |
| 2020/7/2 | noon | DW752 | *Pseudohemiculter dispar* | MZ149081 |
| 2020/7/2 | noon | DW755 | *Squalidus argentatus* | MZ149240 |
| 2020/7/2 | noon | DW757 | *Pseudohemiculter dispar* | MZ149082 |
| 2020/7/2 | noon | DW758 | *Pseudohemiculter dispar* | MZ149083 |
| 2020/7/2 | noon | DW759 | *Squalidus argentatus* | MZ149241 |
| 2020/7/2 | noon | DW760 | *Squalidus argentatus* | MZ149242 |
| 2020/7/2 | noon | DW761 | *Pseudohemiculter dispar* | MZ149084 |
| 2020/7/2 | noon | DW762 | *Pseudohemiculter dispar* | MZ149085 |
| 2020/7/2 | noon | DW763 | *Pseudohemiculter dispar* | MZ149086 |
| 2020/7/2 | noon | DW766 | *Pseudohemiculter dispar* | MZ149087 |
| 2020/7/2 | night | DW788 | *Squalidus argentatus* | MZ149243 |
| 2020/7/2 | night | DW789 | *Pseudohemiculter dispar* | MZ149088 |
| 2020/7/2 | night | DW790 | *Pseudohemiculter dispar* | MZ149089 |
| 2020/7/2 | night | DW791 | *Pseudohemiculter dispar* | MZ149090 |
| 2020/7/2 | night | DW792 | *Pseudohemiculter dispar* | MZ149091 |
| 2020/7/2 | night | DW794 | *Pseudohemiculter dispar* | MZ149092 |
| 2020/7/2 | night | DW795 | *Pseudohemiculter dispar* | MZ149093 |
| 2020/7/2 | night | DW796 | *Pseudohemiculter dispar* | MZ149094 |
| 2020/7/2 | night | DW797 | *Pseudohemiculter dispar* | MZ149095 |
| 2020/7/2 | night | DW798 | *Pseudohemiculter dispar* | MZ149096 |
| 2020/7/2 | night | DW802 | *Pseudohemiculter dispar* | MZ149098 |
| 2020/7/2 | night | DW804 | *Pseudohemiculter dispar* | MZ149099 |
| 2020/7/2 | night | DW805 | *Pseudohemiculter dispar* | MZ149100 |
| 2020/7/2 | night | DW806 | *Pseudohemiculter dispar* | MZ149101 |
| 2020/7/2 | night | DW807 | *Pseudohemiculter dispar* | MZ149102 |
| 2020/7/2 | night | DW808 | *Pseudohemiculter dispar* | MZ149103 |
| 2020/7/2 | night | DW809 | *Pseudohemiculter dispar* | MZ149104 |
| 2020/7/2 | night | DW810 | *Gobiobotia meridionalis* | MZ148896 |
| 2020/7/2 | night | DW812 | *Pseudohemiculter dispar* | MZ149105 |
| 2020/7/2 | night | DW813 | *Pseudohemiculter dispar* | MZ149106 |
| 2020/7/2 | night | DW814 | *Pseudohemiculter dispar* | MZ149107 |
| 2020/7/2 | night | DW815 | *Pseudohemiculter dispar* | MZ149108 |
| 2020/7/2 | night | DW816 | *Pseudohemiculter dispar* | MZ149109 |
| 2020/7/2 | night | DW817 | *Pseudohemiculter dispar* | MZ149110 |
| 2020/7/2 | night | DW818 | *Pseudohemiculter dispar* | MZ149111 |
| 2020/7/2 | night | DW819 | *Pseudohemiculter dispar* | MZ149112 |
| 2020/7/2 | night | DW820 | *Pseudohemiculter dispar* | MZ149113 |
| 2020/7/2 | night | DW821 | *Pseudohemiculter dispar* | MZ149114 |
| 2020/7/2 | night | DW825 | *Pseudohemiculter dispar* | MZ149115 |
| 2020/7/2 | night | DW827 | *Pseudohemiculter dispar* | MZ149116 |
| 2020/7/2 | night | DW828 | *Pseudohemiculter dispar* | MZ149117 |
| 2020/7/2 | night | DW829 | *Pseudohemiculter dispar* | MZ149118 |
| 2020/7/2 | night | DW830 | *Pseudohemiculter dispar* | MZ149119 |
| 2020/7/2 | night | DW831 | *Pseudohemiculter dispar* | MZ149120 |
| 2020/7/2 | night | DW832 | *Pseudohemiculter dispar* | MZ149121 |
| 2020/7/2 | night | DW838 | *Pseudohemiculter dispar* | MZ149122 |
| 2020/7/2 | night | DW839 | *Squalidus argentatus* | MZ149244 |
| 2020/7/2 | night | DW840 | *Pseudohemiculter dispar* | MZ149123 |
| 2020/7/2 | night | DW841 | *Pseudohemiculter dispar* | MZ149124 |
| 2020/7/2 | night | DW842 | *Pseudohemiculter dispar* | MZ149125 |
| 2020/7/2 | night | DW844 | *Pseudohemiculter dispar* | MZ149126 |
| 2020/7/2 | night | DW851 | *Squalidus argentatus* | MZ149245 |
| 2020/7/2 | night | DW854 | *Pseudohemiculter dispar* | MZ149127 |
| 2020/7/2 | night | DW857 | *Pseudohemiculter dispar* | MZ149128 |
| 2020/7/2 | night | DW876 | *Gobiobotia meridionalis* | MZ148897 |
| 2020/7/10 | morning | DW916 | *Gobiobotia meridionalis* | MZ148898 |
| 2020/7/10 | noon | DW927 | *Gobiobotia meridionalis* | MZ148899 |
| 2020/7/10 | noon | DW928 | *Pseudohemiculter dispar* | MZ149130 |
| 2020/7/10 | noon | DW929 | *Squalidus argentatus* | MZ149246 |
| 2020/7/10 | noon | DW931 | *Gobiobotia meridionalis* | MZ148900 |
| 2020/7/10 | night | DW932 | *Sinibotia pulchra* | MZ149173 |
| 2020/7/10 | night | DW933 | *Gobiobotia meridionalis* | MZ148901 |
| 2020/7/18 | morning | DW934 | *Pseudohemiculter dispar* | MZ149131 |
| 2020/7/18 | morning | DW935 | *Pseudohemiculter dispar* | MZ149132 |
| 2020/7/18 | morning | DW936 | *Pseudohemiculter dispar* | MZ149133 |
| 2020/7/18 | morning | DW937 | *Pseudohemiculter dispar* | MZ149134 |
| 2020/7/18 | morning | DW938 | *Pseudohemiculter dispar* | MZ149135 |
| 2020/7/18 | morning | DW939 | *Pseudohemiculter dispar* | MZ149136 |
| 2020/7/18 | morning | DW940 | *Pseudohemiculter dispar* | MZ149137 |
| 2020/7/18 | morning | DW941 | *Pseudohemiculter dispar* | MZ149138 |
| 2020/7/18 | morning | DW942 | *Gobiobotia meridionalis* | MZ148902 |
| 2020/7/18 | morning | DW943 | *Pseudohemiculter dispar* | MZ149139 |
| 2020/7/18 | morning | DW944 | *Pseudohemiculter dispar* | MZ149140 |
| 2020/7/18 | morning | DW945 | *Pseudohemiculter dispar* | MZ149141 |
| 2020/7/18 | morning | DW946 | *Pseudohemiculter dispar* | MZ149142 |
| 2020/7/18 | morning | DW948 | *Pseudohemiculter dispar* | MZ149143 |
| 2020/7/18 | morning | DW949 | *Pseudohemiculter dispar* | MZ149144 |
| 2020/7/18 | morning | DW950 | *Pseudohemiculter dispar* | MZ149145 |
| 2020/7/18 | morning | DW951 | *Pseudohemiculter dispar* | MZ149146 |
| 2020/7/18 | morning | DW952 | *Pseudohemiculter dispar* | MZ149147 |
| 2020/7/18 | morning | DW954 | *Pseudohemiculter dispar* | MZ149148 |
| 2020/7/18 | morning | DW955 | *Pseudohemiculter dispar* | MZ149149 |
| 2020/7/18 | morning | DW956 | *Pseudohemiculter dispar* | MZ149150 |
| 2020/7/18 | morning | DW957 | *Pseudohemiculter dispar* | MZ149151 |
| 2020/7/18 | morning | DW958 | *Pseudohemiculter dispar* | MZ149152 |
| 2020/7/18 | morning | DW961 | *Pseudohemiculter dispar* | MZ149153 |
| 2020/7/18 | morning | DW962 | *Pseudohemiculter dispar* | MZ149154 |
| 2020/7/18 | morning | DW964 | *Pseudohemiculter dispar* | MZ149155 |
| 2020/7/18 | morning | DW966 | *Pseudohemiculter dispar* | MZ149156 |
| 2020/7/18 | morning | DW968 | *Pseudohemiculter dispar* | MZ149157 |
| 2020/7/18 | morning | DW969 | *Pseudohemiculter dispar* | MZ149158 |
| 2020/7/18 | morning | DW971 | *Pseudohemiculter dispar* | MZ149159 |
| 2020/7/18 | morning | DW978 | *Pseudohemiculter dispar* | MZ149160 |
| 2020/7/18 | morning | DW981 | *Gobiobotia meridionalis* | MZ148903 |
| 2020/7/18 | morning | DW983 | *Squalidus argentatus* | MZ149247 |
| 2020/7/18 | morning | DW984 | *Pseudohemiculter dispar* | MZ149161 |
| 2020/7/18 | morning | DW985 | *Pseudohemiculter dispar* | MZ149162 |
| 2020/7/18 | morning | DW986 | *Pseudohemiculter dispar* | MZ149163 |
| 2020/7/18 | morning | DW1035 | *Squalidus argentatus* | MZ149220 |
| 2020/7/18 | noon | DW1088 | *Sinibotia pulchra* | MZ149167 |
| 2020/7/18 | noon | DW1124 | *Sinibotia robusta* | MZ149176 |
| 2020/7/18 | noon | DW1140 | *Pseudohemiculter dispar* | MZ148921 |
| 2020/7/18 | noon | DW1142 | *Pseudohemiculter dispar* | MZ148922 |
| 2020/7/18 | noon | DW1145 | *Pseudohemiculter dispar* | MZ148923 |
| 2020/7/18 | noon | DW1146 | *Squalidus argentatus* | MZ149221 |
| 2020/7/18 | noon | DW1147 | *Pseudohemiculter dispar* | MZ148924 |
| 2020/7/18 | noon | DW1148 | *Pseudohemiculter dispar* | MZ148925 |
| 2020/7/18 | noon | DW1149 | *Pseudohemiculter dispar* | MZ148926 |
| 2020/7/18 | noon | DW1150 | *Pseudohemiculter dispar* | MZ148928 |
| 2020/7/18 | noon | DW1152 | *Pseudohemiculter dispar* | MZ148929 |
| 2020/7/18 | noon | DW1153 | *Pseudohemiculter dispar* | MZ148930 |
| 2020/7/18 | noon | DW1154 | *Pseudohemiculter dispar* | MZ148931 |
| 2020/7/18 | noon | DW1155 | *Pseudohemiculter dispar* | MZ148932 |
| 2020/7/18 | noon | DW1156 | *Pseudohemiculter dispar* | MZ148933 |
| 2020/7/18 | noon | DW1157 | *Pseudohemiculter dispar* | MZ148934 |
| 2020/7/18 | noon | DW1158 | *Pseudohemiculter dispar* | MZ148935 |
| 2020/7/18 | noon | DW1161 | *Pseudohemiculter dispar* | MZ148937 |
| 2020/7/18 | noon | DW1162 | *Pseudohemiculter dispar* | MZ148938 |
| 2020/7/18 | noon | DW1164 | *Pseudohemiculter dispar* | MZ148939 |
| 2020/7/18 | noon | DW1165 | *Pseudohemiculter dispar* | MZ148940 |
| 2020/7/18 | noon | DW1166 | *Pseudohemiculter dispar* | MZ148941 |
| 2020/7/18 | night | DW1168 | *Pseudohemiculter dispar* | MZ148942 |
| 2020/7/18 | night | DW1171 | *Pseudohemiculter dispar* | MZ148944 |
| 2020/7/18 | night | DW1173 | *Pseudohemiculter dispar* | MZ148945 |
| 2020/7/18 | night | DW1176 | *Pseudohemiculter dispar* | MZ148946 |
| 2020/7/18 | night | DW1179 | *Squalidus argentatus* | MZ149222 |
| 2020/7/18 | night | DW1181 | *Pseudohemiculter dispar* | MZ148947 |
| 2020/7/18 | night | DW1182 | *Pseudohemiculter dispar* | MZ148948 |
| 2020/7/18 | night | DW1189 | *Pseudohemiculter dispar* | MZ148949 |
| 2020/7/18 | night | DW1192 | *Pseudohemiculter dispar* | MZ148950 |
| 2020/7/18 | night | DW1203 | *Sinibotia pulchra* | MZ149168 |
| 2020/7/18 | night | DW1212 | *Pseudohemiculter dispar* | MZ148952 |
| 2020/8/2 | morning | DW1217 | *Squalidus argentatus* | MZ149223 |
| 2020/8/2 | morning | DW1218 | *Squalidus argentatus* | MZ149224 |
| 2020/8/2 | noon | DW1224 | *Pseudohemiculter dispar* | MZ148953 |
| 2020/8/2 | noon | DW1227 | *Pseudohemiculter dispar* | MZ148954 |
| 2020/8/10 | morning | DW1235 | *Pseudohemiculter dispar* | MZ148955 |
| 2020/8/10 | morning | DW1237 | *Pseudohemiculter dispar* | MZ148956 |
| 2020/8/10 | morning | DW1238 | *Pseudohemiculter dispar* | MZ148957 |
| 2020/8/10 | noon | DW1241 | *Squalidus argentatus* | MZ149225 |
| 2020/8/10 | night | DW1244 | *Gobiobotia meridionalis* | MZ148861 |
| 2020/8/10 | night | DW1245 | *Squalidus argentatus* | MZ149226 |
| 2020/8/18 | morning | DW1252 | *Squalidus argentatus* | MZ149227 |
| 2020/8/18 | morning | DW1253 | *Squalidus argentatus* | MZ149228 |
| 2020/8/18 | morning | DW1255 | *Squalidus argentatus* | MZ149229 |
| 2020/8/18 | noon | DW1257 | *Squalidus argentatus* | MZ149230 |
| 2020/8/18 | noon | DW1258 | *Gobiobotia meridionalis* | MZ148862 |
| 2020/8/18 | noon | DW1259 | *Squalidus argentatus* | MZ149231 |
| 2020/8/18 | noon | DW1260 | *Squalidus argentatus* | MZ149232 |
| 2020/8/18 | noon | DW1262 | *Squalidus argentatus* | MZ149233 |
| 2020/8/18 | night | DW1264 | *Squalidus argentatus* | MZ149234 |
| 2020/8/18 | night | DW1265 | *Squalidus argentatus* | MZ149235 |
| 2020/8/26 | morning | DW1267 | *Sinibotia robusta* | MZ149180 |
| 2020/8/26 | morning | DW1268 | *Sinibotia robusta* | MZ149181 |
| 2020/8/26 | morning | DW1269 | *Sinibotia robusta* | MZ149182 |
| 2020/8/26 | morning | DW1270 | *Sinibotia robusta* | MZ149183 |
| 2020/8/26 | morning | DW1271 | *Sinibotia robusta* | MZ149184 |
| 2020/8/26 | morning | DW1272 | *Sinibotia robusta* | MZ149185 |
| 2020/8/26 | morning | DW1273 | *Sinibotia robusta* | MZ149186 |
| 2020/8/26 | morning | DW1274 | *Sinibotia robusta* | MZ149187 |
| 2020/8/26 | morning | DW1275 | *Sinibotia robusta* | MZ149188 |
| 2020/8/26 | morning | DW1276 | *Sinibotia robusta* | MZ149189 |
| 2020/8/26 | morning | DW1277 | *Sinibotia robusta* | MZ149190 |
| 2020/8/26 | morning | DW1278 | *Sinibotia pulchra* | MZ149169 |
| 2020/8/26 | morning | DW1279 | *Sinibotia pulchra* | MZ149170 |
| 2020/8/26 | morning | DW1280 | *Sinibotia robusta* | MZ149191 |
| 2020/8/26 | morning | DW1281 | *Sinibotia robusta* | MZ149192 |
| 2020/8/26 | morning | DW1282 | *Sinibotia robusta* | MZ149193 |
| 2020/8/26 | noon | DW1283 | *Squaliobarbus curriculus* | MZ149248 |
| 2020/9/8 | morning | DW1284 | *Sinibotia pulchra* | MZ149171 |
| 2020/5/18 | morning | DWZY2* | *Onychostoma gerlachi* | MZ148907 |
| 2020/6/2 | morning | DWZY3* | *Siniperca scherzeri* | MZ149214 |
| 2020/6/18 | morning | DWZY4* | *Pseudohemiculter dispar* | MZ149164 |
| 2020/6/26 | morning | DWZY5* | *Onychostoma gerlachi* | MZ148908 |
| 2020/6/26 | night | DWZY6* | *Onychostoma gerlachi* | MZ148909 |
| 2020/6/26 | night | DWZY7* | *Onychostoma gerlachi* | MZ148910 |
| 2020/7/10 | morning | DWZY8* | *Rhinogobius* sp.2 | / |
| 2020/7/10 | morning | DWZY9* | *Rhinogobius* sp.1 | / |
| 2020/7/10 | morning | DWZY10* | *Siniperca scherzeri* | MZ149211 |
| 2020/7/18 | morning | DWZY11* | *Siniperca scherzeri* | MZ149212 |
| 2020/7/18 | morning | DWZY12* | Unknown species 2 | / |
| 2020/7/18 | morning | DWZY13* | *Rhinogobius* sp.2 | / |
| 2020/8/2 | noon | DWZY14* | *Siniperca scherzeri* | MZ149213 |
| 2020/8/2 | noon | DWZY15* | *Zacco platypus* | MZ149250 |
| 2020/8/2 | night | DWZY16* | *Onychostoma gerlachi* | MZ148905 |
| 2020/8/2 | night | DWZY17* | *Mastacembelus armatus* | MZ148904 |
| 2020/8/26 | night | DWZY18* | *Onychostoma gerlachi* | MZ148906 |
