# Supplementary material for: DNA barcoding reveals the temporal community composition of drifting fish eggs in the lower Hongshui River, China
Source: Ecol Evol. 2021 Jul 22;11(16):11507–14. doi: 10.1002/ece3.7943 (PMC8366882; doi:10.1002/ece3.7943)
Supplement: Supplementary file 2 — Table S2 [file ECE3-11-11507-s004.docx]

| Sample ID | Final name | Best match species | Genbank Nos | Simililarity | Nearest species | Genbank Nos | Simililarity |
| --- | --- | --- | --- | --- | --- | --- | --- |
| DW0091 | *Pseudohemiculter dispar* | *Pseudohemiculter dispar* | Unpublished | 99.82 | *Hemiculterella sauvagei* | Unpublished | 92.11 |
| DW0094 | *Gobiobotia meridionalis* | *Gobiobotia meridionalis* | JN003344 | 99.65 | *Gobiobotia naktongensis* | NC020464 | 89.36 |
| DW0097 | *Pseudohemiculter dispar* | *Pseudohemiculter dispar* | Unpublished | 100 | *Hemiculterella sauvagei* | Unpublished | 91.76 |
| DW0099 | *Pseudohemiculter dispar* | *Pseudohemiculter dispar* | Unpublished | 100 | *Hemiculterella sauvagei* | Unpublished | 91.76 |
| DW0101 | *Pseudohemiculter dispar* | *Pseudohemiculter dispar* | Unpublished | 100 | *Hemiculterella sauvagei* | Unpublished | 91.94 |
| DW0103 | *Pseudohemiculter dispar* | *Pseudohemiculter dispar* | Unpublished | 100 | *Hemiculterella sauvagei* | Unpublished | 91.76 |
| DW0104 | *Sinibotia robusta* | *Sinibotia robusta* | KT374012 | 100 | *Sinibotia reevesae* | MH027676 | 91.84 |
| DW0105 | *Pseudohemiculter dispar* | *Pseudohemiculter dispar* | Unpublished | 100 | *Hemiculterella sauvagei* | Unpublished | 91.76 |
| DW0106 | *Sinibotia robusta* | *Sinibotia robusta* | KT374012 | 100 | *Sinibotia reevesae* | MH027676 | 91.84 |
| DW0134 | *Pseudohemiculter dispar* | *Pseudohemiculter dispar* | Unpublished | 100 | *Hemiculterella sauvagei* | Unpublished | 91.76 |
| DW1 | *Pseudohemiculter dispar* | *Pseudohemiculter dispar* | Unpublished | 100 | *Hemiculterella sauvagei* | Unpublished | 91.76 |
| DW10 | *Pseudohemiculter dispar* | *Pseudohemiculter dispar* | Unpublished | 100 | *Hemiculterella sauvagei* | Unpublished | 91.58 |
| DW1035 | *Squalidus argentatus* | *Squalidus argentatus* | Unpublished | 99.82 | *Squalidus chankaensis* | Unpublished | 98.58 |
| DW1088 | *Sinibotia pulchra* | *Sinibotia pulchra* | MH027667 | 100 | *Sinibotia superciliaris* | MH027682 | 90.6 |
| DW11 | *Pseudohemiculter dispar* | *Pseudohemiculter dispar* | Unpublished | 100 | *Hemiculterella sauvagei* | Unpublished | 91.76 |
| DW111 | *Gobiobotia meridionalis* | *Gobiobotia meridionalis* | JN003344 | 99.65 | *Gobiobotia naktongensis* | NC020464 | 89.36 |
| DW1124 | *Sinibotia robusta* | *Sinibotia robusta* | MH027667 | 100 | *Sinibotia reevesae* | MH027676 | 92.2 |
| DW113 | *Sinibotia robusta* | *Sinibotia robusta* | MH027665 | 99.82 | *Sinibotia reevesae* | MH027676 | 91.84 |
| DW114 | *Sinogastromyzon wui* | *Sinogastromyzon wui* | JN177077 | 99.47 | *Lepturichthys fimbriata* | JN830340 | 94.86 |
| DW1140 | *Pseudohemiculter dispar* | *Pseudohemiculter dispar* | Unpublished | 100 | *Hemiculterella sauvagei* | Unpublished | 91.76 |
| DW1142 | *Pseudohemiculter dispar* | *Pseudohemiculter dispar* | Unpublished | 99.82 | *Hemiculterella sauvagei* | Unpublished | 92.11 |
| DW1145 | *Pseudohemiculter dispar* | *Pseudohemiculter dispar* | Unpublished | 100 | *Hemiculterella sauvagei* | Unpublished | 91.76 |
| DW1146 | *Squalidus argentatus* | *Squalidus argentatus* | Unpublished | 100 | *Squalidus chankaensis* | Unpublished | 98.76 |
| DW1147 | *Pseudohemiculter dispar* | *Pseudohemiculter dispar* | Unpublished | 100 | *Hemiculterella sauvagei* | Unpublished | 91.76 |
| DW1148 | *Pseudohemiculter dispar* | *Pseudohemiculter dispar* | Unpublished | 100 | *Hemiculterella sauvagei* | Unpublished | 91.76 |
| DW1149 | *Pseudohemiculter dispar* | *Pseudohemiculter dispar* | Unpublished | 99.82 | *Hemiculterella sauvagei* | Unpublished | 91.76 |
| DW115 | *Pseudohemiculter dispar* | *Pseudohemiculter dispar* | Unpublished | 100 | *Hemiculterella sauvagei* | Unpublished | 91.76 |
| DW1150 | *Pseudohemiculter dispar* | *Pseudohemiculter dispar* | Unpublished | 100 | *Hemiculterella sauvagei* | Unpublished | 91.76 |
| DW1152 | *Pseudohemiculter dispar* | *Pseudohemiculter dispar* | Unpublished | 100 | *Hemiculterella sauvagei* | Unpublished | 91.76 |
| DW1153 | *Pseudohemiculter dispar* | *Pseudohemiculter dispar* | Unpublished | 99.82 | *Hemiculterella sauvagei* | Unpublished | 91.76 |
| DW1154 | *Pseudohemiculter dispar* | *Pseudohemiculter dispar* | Unpublished | 100 | *Hemiculterella sauvagei* | Unpublished | 91.76 |
| DW1155 | *Pseudohemiculter dispar* | *Pseudohemiculter dispar* | Unpublished | 100 | *Hemiculterella sauvagei* | Unpublished | 91.76 |
| DW1156 | *Pseudohemiculter dispar* | *Pseudohemiculter dispar* | Unpublished | 100 | *Hemiculterella sauvagei* | Unpublished | 91.76 |
| DW1157 | *Pseudohemiculter dispar* | *Pseudohemiculter dispar* | Unpublished | 100 | *Hemiculterella sauvagei* | Unpublished | 91.76 |
| DW1158 | *Pseudohemiculter dispar* | *Pseudohemiculter dispar* | Unpublished | 100 | *Hemiculterella sauvagei* | Unpublished | 91.76 |
| DW116 | *Pseudohemiculter dispar* | *Pseudohemiculter dispar* | Unpublished | 100 | *Hemiculterella sauvagei* | Unpublished | 91.76 |
| DW1161 | *Pseudohemiculter dispar* | *Pseudohemiculter dispar* | Unpublished | 99.82 | *Hemiculterella sauvagei* | Unpublished | 92.11 |
| DW1162 | *Pseudohemiculter dispar* | *Pseudohemiculter dispar* | Unpublished | 100 | *Hemiculterella sauvagei* | Unpublished | 91.76 |
| DW1164 | *Pseudohemiculter dispar* | *Pseudohemiculter dispar* | Unpublished | 100 | *Hemiculterella sauvagei* | Unpublished | 91.76 |
| DW1165 | *Pseudohemiculter dispar* | *Pseudohemiculter dispar* | Unpublished | 100 | *Hemiculterella sauvagei* | Unpublished | 91.76 |
| DW1166 | *Pseudohemiculter dispar* | *Pseudohemiculter dispar* | Unpublished | 100 | *Hemiculterella sauvagei* | Unpublished | 91.76 |
| DW1168 | *Pseudohemiculter dispar* | *Pseudohemiculter dispar* | Unpublished | 100 | *Hemiculterella sauvagei* | Unpublished | 91.76 |
| DW117 | *Pseudohemiculter dispar* | *Pseudohemiculter dispar* | Unpublished | 99.82 | *Hemiculterella sauvagei* | Unpublished | 91.58 |
| DW1171 | *Pseudohemiculter dispar* | *Pseudohemiculter dispar* | Unpublished | 100 | *Hemiculterella sauvagei* | Unpublished | 91.94 |
| DW1173 | *Pseudohemiculter dispar* | *Pseudohemiculter dispar* | Unpublished | 99.82 | *Hemiculterella sauvagei* | Unpublished | 91.58 |
| DW1176 | *Pseudohemiculter dispar* | *Pseudohemiculter dispar* | Unpublished | 100 | *Hemiculterella sauvagei* | Unpublished | 91.76 |
| DW1179 | *Squalidus argentatus* | *Squalidus argentatus* | Unpublished | 100 | *Squalidus chankaensis* | Unpublished | 98.76 |
| DW1181 | *Pseudohemiculter dispar* | *Pseudohemiculter dispar* | Unpublished | 100 | *Hemiculterella sauvagei* | Unpublished | 91.58 |
| DW1182 | *Pseudohemiculter dispar* | *Pseudohemiculter dispar* | Unpublished | 100 | *Hemiculterella sauvagei* | Unpublished | 91.76 |
| DW1189 | *Pseudohemiculter dispar* | *Pseudohemiculter dispar* | Unpublished | 100 | *Hemiculterella sauvagei* | Unpublished | 91.58 |
| DW119 | *Sinibotia robusta* | *Sinibotia robusta* | MH027669 | 100 | *Sinibotia reevesae* | MH027676 | 91.67 |
| DW1192 | *Pseudohemiculter dispar* | *Pseudohemiculter dispar* | Unpublished | 100 | *Hemiculterella sauvagei* | Unpublished | 91.76 |
| DW12 | *Pseudohemiculter dispar* | *Pseudohemiculter dispar* | Unpublished | 100 | *Hemiculterella sauvagei* | Unpublished | 91.76 |
| DW120 | *Sinibotia robusta* | *Sinibotia robusta* | MH027668 | 99.82 | *Sinibotia superciliaris* | KC871100 | 91.83 |
| DW1203 | *Sinibotia pulchra* | *Sinibotia pulchra* | MH027666 | 100 | *Sinibotia superciliaris* | MH027682 | 90.6 |
| DW1212 | *Pseudohemiculter dispar* | *Pseudohemiculter dispar* | Unpublished | 100 | *Hemiculterella sauvagei* | Unpublished | 91.76 |
| DW1217 | *Squalidus argentatus* | *Squalidus argentatus* | Unpublished | 100 | *Squalidus chankaensis* | Unpublished | 98.76 |
| DW1218 | *Squalidus argentatus* | *Squalidus argentatus* | Unpublished | 99.82 | *Squalidus chankaensis* | Unpublished | 98.58 |
| DW122 | *Sinogastromyzon wui* | *Sinogastromyzon wui* | JN177077 | 99.47 | *Lepturichthys fimbriata* | JN830340 | 94.86 |
| DW1224 | *Pseudohemiculter dispar* | *Pseudohemiculter dispar* | Unpublished | 100 | *Hemiculterella sauvagei* | Unpublished | 91.76 |
| DW1227 | *Pseudohemiculter dispar* | *Pseudohemiculter dispar* | Unpublished | 100 | *Hemiculterella sauvagei* | Unpublished | 91.76 |
| DW1235 | *Pseudohemiculter dispar* | *Pseudohemiculter dispar* | Unpublished | 100 | *Hemiculterella sauvagei* | Unpublished | 91.76 |
| DW1237 | *Pseudohemiculter dispar* | *Pseudohemiculter dispar* | Unpublished | 99.82 | *Hemiculterella sauvagei* | Unpublished | 91.58 |
| DW1238 | *Pseudohemiculter dispar* | *Pseudohemiculter dispar* | Unpublished | 100 | *Hemiculterella sauvagei* | Unpublished | 91.76 |
| **DW1241** | *Squalidus argentatus* | *Squalidus argentatus* | KR862242 | 99.82 | NA | NA | NA |
| DW1244 | *Gobiobotia meridionalis* | *Gobiobotia meridionalis* | JN003344 | 99.29 | *Gobiobotia naktongensis* | NC020464 | 89.36 |
| **DW1245** | *Squalidus argentatus* | *Squalidus argentatus* | KR862242 | 100 | NA | NA | NA |
| DW125 | *Pseudohemiculter dispar* | *Pseudohemiculter dispar* | Unpublished | 100 | *Hemiculterella sauvagei* | Unpublished | 91.76 |
| **DW1252** | *Squalidus argentatus* | *Squalidus argentatus* | KR862242 | 99.65 | NA | NA | NA |
| **DW1253** | *Squalidus argentatus* | *Squalidus argentatus* | KR862242 | 100 | NA | NA | NA |
| **DW1255** | *Squalidus argentatus* | *Squalidus argentatus* | KR862242 | 100 | NA | NA | NA |
| DW1257 | *Squalidus argentatus* | *Squalidus argentatus* | Unpublished | 99.65 | *Squalidus chankaensis* | Unpublished | 98.40 |
| DW1258 | *Gobiobotia meridionalis* | *Gobiobotia meridionalis* | JN003344 | 99.65 | *Gobiobotia naktongensis* | NC020464 | 89.36 |
| DW1259 | *Squalidus argentatus* | *Squalidus argentatus* | Unpublished | 100 | *Squalidus chankaensis* | Unpublished | 98.76 |
| DW1260 | *Squalidus argentatus* | *Squalidus argentatus* | Unpublished | 100 | *Squalidus chankaensis* | Unpublished | 98.76 |
| DW1262 | *Squalidus argentatus* | *Squalidus argentatus* | Unpublished | 100 | *Squalidus chankaensis* | Unpublished | 98.76 |
| **DW1264** | *Squalidus argentatus* | *Squalidus argentatus* | KR862242 | 100 | NA | NA | NA |
| **DW1265** | *Squalidus argentatus* | *Squalidus argentatus* | KR862242 | 100 | NA | NA | NA |
| DW1267 | *Sinibotia robusta* | *Sinibotia robusta* | KT374012 | 100 | *Sinibotia reevesae* | MH027676 | 91.84 |
| DW1268 | *Sinibotia robusta* | *Sinibotia robusta* | MH027668 | 100 | *Sinibotia superciliaris* | KC871100 | 91.65 |
| DW1269 | *Sinibotia robusta* | *Sinibotia robusta* | KT374012 | 100 | *Sinibotia reevesae* | MH027676 | 91.84 |
| DW127 | *Pseudohemiculter dispar* | *Pseudohemiculter dispar* | Unpublished | 100 | *Hemiculterella sauvagei* | Unpublished | 91.76 |
| DW1270 | *Sinibotia robusta* | *Sinibotia robusta* | MH027670 | 100 | *Sinibotia reevesae* | MH027676 | 91.67 |
| DW1271 | *Sinibotia robusta* | *Sinibotia robusta* | KT374012 | 99.65 | *Sinibotia reevesae* | MH027676 | 91.49 |
| DW1272 | *Sinibotia robusta* | *Sinibotia robusta* | MH027670 | 100 | *Sinibotia reevesae* | MH027676 | 91.67 |
| DW1273 | *Sinibotia robusta* | *Sinibotia robusta* | MH027665 | 100 | *Sinibotia reevesae* | MH027676 | 92.02 |
| DW1274 | *Sinibotia robusta* | *Sinibotia robusta* | KT374012 | 99.65 | *Sinibotia reevesae* | MH027676 | 91.49 |
| DW1275 | *Sinibotia robusta* | *Sinibotia robusta* | MH027668 | 100 | *Sinibotia superciliaris* | KC871100 | 91.65 |
| DW1276 | *Sinibotia robusta* | *Sinibotia robusta* | MH027668 | 100 | *Sinibotia superciliaris* | KC871100 | 91.65 |
| DW1277 | *Sinibotia robusta* | *Sinibotia robusta* | MH027663 | 100 | *Sinibotia reevesae* | MH027676 | 91.49 |
| DW1278 | *Sinibotia pulchra* | *Sinibotia pulchra* | MH027665 | 100 | *Sinibotia superciliaris* | MH027682 | 90.6 |
| DW1279 | *Sinibotia pulchra* | *Sinibotia pulchra* | MH027664 | 100 | *Sinibotia superciliaris* | MH027682 | 90.6 |
| DW128 | *Pseudohemiculter dispar* | *Pseudohemiculter dispar* | Unpublished | 100 | *Hemiculterella sauvagei* | Unpublished | 91.94 |
| DW1280 | *Sinibotia robusta* | *Sinibotia robusta* | KT374012 | 100 | *Sinibotia reevesae* | MH027676 | 91.84 |
| DW1281 | *Sinibotia robusta* | *Sinibotia robusta* | KT374012 | 100 | *Sinibotia reevesae* | MH027676 | 91.84 |
| DW1282 | *Sinibotia robusta* | *Sinibotia robusta* | MH027668 | 99.82 | *Sinibotia superciliaris* | KC871100 | 91.83 |
| DW1283 | *Squaliobarbus curriculus* | *Squaliobarbus curriculus* | KP769816 | 100 | *Elopichthys bambusa* | NC024834 | 94.05 |
| DW1284 | *Sinibotia pulchra* | *Sinibotia pulchra* | MH027662 | 100 | *Sinibotia superciliaris* | MH027682 | 90.6 |
| DW129 | *Pseudohemiculter dispar* | *Pseudohemiculter dispar* | Unpublished | 100 | *Hemiculterella sauvagei* | Unpublished | 91.76 |
| DW130 | *Pseudohemiculter dispar* | *Pseudohemiculter dispar* | Unpublished | 100 | *Hemiculterella sauvagei* | Unpublished | 91.76 |
| DW131 | *Pseudohemiculter dispar* | *Pseudohemiculter dispar* | Unpublished | 100 | *Hemiculterella sauvagei* | Unpublished | 91.76 |
| DW132 | *Pseudohemiculter dispar* | *Pseudohemiculter dispar* | Unpublished | 100 | *Hemiculterella sauvagei* | Unpublished | 91.76 |
| DW135 | *Pseudohemiculter dispar* | *Pseudohemiculter dispar* | Unpublished | 100 | *Hemiculterella sauvagei* | Unpublished | 91.76 |
| DW136 | *Pseudohemiculter dispar* | *Pseudohemiculter dispar* | Unpublished | 100 | *Hemiculterella sauvagei* | Unpublished | 91.76 |
| DW137 | *Gobiobotia meridionalis* | *Gobiobotia meridionalis* | JN003344 | 99.47 | *Gobiobotia naktongensis* | NC020464 | 89.18 |
| DW138 | *Pseudohemiculter dispar* | *Pseudohemiculter dispar* | Unpublished | 100 | *Hemiculterella sauvagei* | Unpublished | 91.76 |
| DW139 | *Sinogastromyzon wui* | *Sinogastromyzon wui* | JN177077 | 99.47 | *Lepturichthys fimbriata* | JN830340 | 94.86 |
| DW140 | *Pseudohemiculter dispar* | *Pseudohemiculter dispar* | Unpublished | 100 | *Hemiculterella sauvagei* | Unpublished | 91.76 |
| DW141 | *Pseudohemiculter dispar* | *Pseudohemiculter dispar* | Unpublished | 100 | *Hemiculterella sauvagei* | Unpublished | 91.76 |
| DW142 | *Pseudohemiculter dispar* | *Pseudohemiculter dispar* | Unpublished | 100 | *Hemiculterella sauvagei* | Unpublished | 91.94 |
| DW143 | *Sinogastromyzon wui* | *Sinogastromyzon wui* | JN177077 | 99.29 | *Lepturichthys fimbriata* | JN830340 | 95.04 |
| DW144 | *Gobiobotia meridionalis* | *Gobiobotia meridionalis* | JN003344 | 100 | *Gobiobotia filifer* | MK834305 | 89.36 |
| DW145 | *Pseudohemiculter dispar* | *Pseudohemiculter dispar* | Unpublished | 100 | *Hemiculterella sauvagei* | Unpublished | 91.94 |
| DW147 | *Pseudohemiculter dispar* | *Pseudohemiculter dispar* | Unpublished | 100 | *Hemiculterella sauvagei* | Unpublished | 91.76 |
| DW148 | *Sinibotia robusta* | *Sinibotia robusta* | KT374012 | 100 | *Sinibotia reevesae* | MH027676 | 91.84 |
| DW149 | *Pseudohemiculter dispar* | *Pseudohemiculter dispar* | Unpublished | 100 | *Hemiculterella sauvagei* | Unpublished | 91.76 |
| DW151 | *Pseudohemiculter dispar* | *Pseudohemiculter dispar* | Unpublished | 100 | *Hemiculterella sauvagei* | Unpublished | 91.76 |
| DW152 | *Gobiobotia meridionalis* | *Gobiobotia meridionalis* | JN003344 | 99.65 | *Gobiobotia naktongensis* | NC020464 | 89.36 |
| DW153 | *Sinibotia robusta* | *Sinibotia robusta* | KT374012 | 100 | *Sinibotia reevesae* | MH027676 | 91.84 |
| DW154 | *Gobiobotia meridionalis* | *Gobiobotia meridionalis* | JN003344 | 100 | *Gobiobotia filifer* | MK834305 | 89.36 |
| DW155 | *Pseudohemiculter dispar* | *Pseudohemiculter dispar* | Unpublished | 100 | *Hemiculterella sauvagei* | Unpublished | 91.76 |
| DW157 | *Pseudohemiculter dispar* | *Pseudohemiculter dispar* | Unpublished | 100 | *Hemiculterella sauvagei* | Unpublished | 91.76 |
| DW158 | *Pseudohemiculter dispar* | *Pseudohemiculter dispar* | Unpublished | 100 | *Hemiculterella sauvagei* | Unpublished | 91.76 |
| DW159 | *Pseudohemiculter dispar* | *Pseudohemiculter dispar* | Unpublished | 100 | *Hemiculterella sauvagei* | Unpublished | 91.76 |
| DW162 | *Gobiobotia meridionalis* | *Gobiobotia meridionalis* | JN003344 | 99.65 | *Gobiobotia naktongensis* | NC020464 | 89.36 |
| DW166 | *Pseudohemiculter dispar* | *Pseudohemiculter dispar* | Unpublished | 100 | *Hemiculterella sauvagei* | Unpublished | 91.94 |
| DW168 | *Sinogastromyzon wui* | *Sinogastromyzon wui* | JN177077 | 99.47 | *Lepturichthys fimbriata* | JN830340 | 94.86 |
| DW170 | *Pseudohemiculter dispar* | *Pseudohemiculter dispar* | Unpublished | 100 | *Hemiculterella sauvagei* | Unpublished | 91.76 |
| DW171 | *Pseudohemiculter dispar* | *Pseudohemiculter dispar* | Unpublished | 100 | *Hemiculterella sauvagei* | Unpublished | 91.76 |
| DW172 | *Pseudohemiculter dispar* | *Pseudohemiculter dispar* | Unpublished | 100 | *Hemiculterella sauvagei* | Unpublished | 91.76 |
| DW173 | *Pseudohemiculter dispar* | *Pseudohemiculter dispar* | Unpublished | 100 | *Hemiculterella sauvagei* | Unpublished | 91.76 |
| DW176 | *Pseudohemiculter dispar* | *Pseudohemiculter dispar* | Unpublished | 99.82 | *Hemiculterella sauvagei* | Unpublished | 91.76 |
| DW178 | *Pseudohemiculter dispar* | *Pseudohemiculter dispar* | Unpublished | 99.82 | *Hemiculterella sauvagei* | Unpublished | 91.58 |
| DW180 | *Pseudohemiculter dispar* | *Pseudohemiculter dispar* | Unpublished | 100 | *Hemiculterella sauvagei* | Unpublished | 91.76 |
| DW186 | *Pseudohemiculter dispar* | *Pseudohemiculter dispar* | Unpublished | 100 | *Hemiculterella sauvagei* | Unpublished | 91.76 |
| DW187 | *Pseudohemiculter dispar* | *Pseudohemiculter dispar* | Unpublished | 100 | *Hemiculterella sauvagei* | Unpublished | 91.76 |
| DW188 | *Pseudohemiculter dispar* | *Pseudohemiculter dispar* | Unpublished | 100 | *Hemiculterella sauvagei* | Unpublished | 91.76 |
| DW189 | *Sinibotia robusta* | *Sinibotia robusta* | MH027665 | 100 | *Sinibotia reevesae* | MH027676 | 92.02 |
| DW191 | *Pseudohemiculter dispar* | *Pseudohemiculter dispar* | Unpublished | 100 | *Hemiculterella sauvagei* | Unpublished | 91.76 |
| DW193 | *Pseudohemiculter dispar* | *Pseudohemiculter dispar* | Unpublished | 100 | *Hemiculterella sauvagei* | Unpublished | 91.94 |
| DW194 | *Pseudohemiculter dispar* | *Pseudohemiculter dispar* | Unpublished | 100 | *Hemiculterella sauvagei* | Unpublished | 91.76 |
| DW195 | *Pseudohemiculter dispar* | *Pseudohemiculter dispar* | Unpublished | 100 | *Hemiculterella sauvagei* | Unpublished | 91.76 |
| DW196 | *Pseudohemiculter dispar* | *Pseudohemiculter dispar* | Unpublished | 100 | *Hemiculterella sauvagei* | Unpublished | 91.76 |
| DW197 | *Pseudohemiculter dispar* | *Pseudohemiculter dispar* | Unpublished | 100 | *Hemiculterella sauvagei* | Unpublished | 91.76 |
| DW198 | *Pseudohemiculter dispar* | *Pseudohemiculter dispar* | Unpublished | 100 | *Hemiculterella sauvagei* | Unpublished | 91.76 |
| DW199 | *Pseudohemiculter dispar* | *Pseudohemiculter dispar* | Unpublished | 100 | *Hemiculterella sauvagei* | Unpublished | 91.76 |
| DW2 | *Pseudohemiculter dispar* | *Pseudohemiculter dispar* | Unpublished | 100 | *Hemiculterella sauvagei* | Unpublished | 91.76 |
| DW200 | *Pseudohemiculter dispar* | *Pseudohemiculter dispar* | Unpublished | 100 | *Hemiculterella sauvagei* | Unpublished | 91.76 |
| DW201 | *Pseudohemiculter dispar* | *Pseudohemiculter dispar* | Unpublished | 100 | *Hemiculterella sauvagei* | Unpublished | 91.76 |
| DW202 | *Pseudohemiculter dispar* | *Pseudohemiculter dispar* | Unpublished | 99.82 | *Hemiculterella sauvagei* | Unpublished | 92.11 |
| DW203 | *Pseudohemiculter dispar* | *Pseudohemiculter dispar* | Unpublished | 100 | *Hemiculterella sauvagei* | Unpublished | 91.94 |
| DW206 | *Pseudohemiculter dispar* | *Pseudohemiculter dispar* | Unpublished | 100 | *Hemiculterella sauvagei* | Unpublished | 91.94 |
| DW207 | *Pseudohemiculter dispar* | *Pseudohemiculter dispar* | Unpublished | 100 | *Hemiculterella sauvagei* | Unpublished | 91.76 |
| DW208 | *Pseudohemiculter dispar* | *Pseudohemiculter dispar* | Unpublished | 100 | *Hemiculterella sauvagei* | Unpublished | 91.76 |
| DW209 | *Pseudohemiculter dispar* | *Pseudohemiculter dispar* | Unpublished | 100 | *Hemiculterella sauvagei* | Unpublished | 91.94 |
| DW210 | *Pseudohemiculter dispar* | *Pseudohemiculter dispar* | Unpublished | 100 | *Hemiculterella sauvagei* | Unpublished | 91.94 |
| DW211 | *Pseudohemiculter dispar* | *Pseudohemiculter dispar* | Unpublished | 100 | *Hemiculterella sauvagei* | Unpublished | 91.76 |
| DW212 | *Pseudohemiculter dispar* | *Pseudohemiculter dispar* | Unpublished | 100 | *Hemiculterella sauvagei* | Unpublished | 91.76 |
| **DW213** | *Pseudolaubuca sinensis* | *Pseudolaubuca sinensis* | Unpublished | 100 | NA | NA | NA |
| DW214 | *Pseudohemiculter dispar* | *Pseudohemiculter dispar* | Unpublished | 100 | *Hemiculterella sauvagei* | Unpublished | 91.76 |
| DW215 | *Pseudohemiculter dispar* | *Pseudohemiculter dispar* | Unpublished | 100 | *Hemiculterella sauvagei* | Unpublished | 91.76 |
| DW216 | *Pseudohemiculter dispar* | *Pseudohemiculter dispar* | Unpublished | 100 | *Hemiculterella sauvagei* | Unpublished | 91.76 |
| DW217 | *Pseudohemiculter dispar* | *Pseudohemiculter dispar* | Unpublished | 100 | *Hemiculterella sauvagei* | Unpublished | 91.76 |
| DW218 | *Pseudohemiculter dispar* | *Pseudohemiculter dispar* | Unpublished | 100 | *Hemiculterella sauvagei* | Unpublished | 91.94 |
| DW219 | *Pseudohemiculter dispar* | *Pseudohemiculter dispar* | Unpublished | 100 | *Hemiculterella sauvagei* | Unpublished | 91.76 |
| DW220 | *Pseudohemiculter dispar* | *Pseudohemiculter dispar* | Unpublished | 100 | *Hemiculterella sauvagei* | Unpublished | 91.76 |
| DW221 | *Pseudohemiculter dispar* | *Pseudohemiculter dispar* | Unpublished | 100 | *Hemiculterella sauvagei* | Unpublished | 91.76 |
| DW223 | *Pseudohemiculter dispar* | *Pseudohemiculter dispar* | Unpublished | 100 | *Hemiculterella sauvagei* | Unpublished | 91.76 |
| DW224 | *Pseudohemiculter dispar* | *Pseudohemiculter dispar* | Unpublished | 99.82 | *Hemiculterella sauvagei* | Unpublished | 91.71 |
| DW225 | *Pseudohemiculter dispar* | *Pseudohemiculter dispar* | Unpublished | 100 | *Hemiculterella sauvagei* | Unpublished | 91.76 |
| DW226 | *Pseudohemiculter dispar* | *Pseudohemiculter dispar* | Unpublished | 100 | *Hemiculterella sauvagei* | Unpublished | 91.94 |
| DW227 | *Pseudohemiculter dispar* | *Pseudohemiculter dispar* | Unpublished | 99.82 | *Hemiculterella sauvagei* | Unpublished | 92.11 |
| DW228 | *Pseudohemiculter dispar* | *Pseudohemiculter dispar* | Unpublished | 100 | *Hemiculterella sauvagei* | Unpublished | 91.76 |
| DW229 | *Pseudohemiculter dispar* | *Pseudohemiculter dispar* | Unpublished | 100 | *Hemiculterella sauvagei* | Unpublished | 91.76 |
| DW230 | *Pseudohemiculter dispar* | *Pseudohemiculter dispar* | Unpublished | 100 | *Hemiculterella sauvagei* | Unpublished | 91.76 |
| **DW231** | *Pseudolaubuca sinensis* | *Pseudolaubuca sinensis* | Unpublished | 100 | NA | NA | NA |
| DW234 | *Pseudohemiculter dispar* | *Pseudohemiculter dispar* | Unpublished | 99.82 | *Hemiculterella sauvagei* | Unpublished | 91.89 |
| DW237 | *Pseudohemiculter dispar* | *Pseudohemiculter dispar* | Unpublished | 99.82 | *Hemiculterella sauvagei* | Unpublished | 91.89 |
| DW238 | *Pseudohemiculter dispar* | *Pseudohemiculter dispar* | Unpublished | 100 | *Hemiculterella sauvagei* | Unpublished | 91.76 |
| DW239 | *Pseudohemiculter dispar* | *Pseudohemiculter dispar* | Unpublished | 99.82 | *Hemiculterella sauvagei* | Unpublished | 91.58 |
| DW242 | *Pseudohemiculter dispar* | *Pseudohemiculter dispar* | Unpublished | 99.82 | *Hemiculterella sauvagei* | Unpublished | 91.58 |
| DW243 | *Pseudohemiculter dispar* | *Pseudohemiculter dispar* | Unpublished | 99.82 | *Hemiculterella sauvagei* | Unpublished | 91.58 |
| DW244 | *Pseudohemiculter dispar* | *Pseudohemiculter dispar* | Unpublished | 100 | *Hemiculterella sauvagei* | Unpublished | 91.62 |
| DW3 | *Pseudohemiculter dispar* | *Pseudohemiculter dispar* | Unpublished | 100 | *Hemiculterella sauvagei* | Unpublished | 91.94 |
| DW300 | *Sinibotia robusta* | *Sinibotia robusta* | KT374012 | 99.82 | *Sinibotia reevesae* | MH027676 | 91.67 |
| DW326 | *Sinibotia robusta* | *Sinibotia robusta* | KT374012 | 100 | *Sinibotia reevesae* | MH027676 | 91.84 |
| DW4 | *Pseudohemiculter dispar* | *Pseudohemiculter dispar* | Unpublished | 99.82 | *Hemiculterella sauvagei* | Unpublished | 91.67 |
| DW41 | *Pseudohemiculter dispar* | *Pseudohemiculter dispar* | Unpublished | 100 | *Hemiculterella sauvagei* | Unpublished | 91.76 |
| DW414 | *Squalidus argentatus* | *Squalidus argentatus* | Unpublished | 100 | *Squalidus chankaensis* | Unpublished | 98.76 |
| DW439 | *Gobiobotia meridionalis* | *Gobiobotia meridionalis* | JN003344 | 99.65 | *Gobiobotia naktongensis* | NC020464 | 89.36 |
| DW440 | *Squalidus argentatus* | *Squalidus argentatus* | Unpublished | 100 | *Squalidus chankaensis* | Unpublished | 98.76 |
| DW442 | *Gobiobotia meridionalis* | *Gobiobotia meridionalis* | JN003344 | 99.65 | *Gobiobotia naktongensis* | NC020464 | 89.36 |
| DW443 | *Gobiobotia meridionalis* | *Gobiobotia meridionalis* | JN003344 | 99.65 | *Gobiobotia naktongensis* | NC020464 | 89.36 |
| DW448 | *Gobiobotia meridionalis* | *Gobiobotia meridionalis* | JN003344 | 99.65 | *Gobiobotia naktongensis* | NC020464 | 89.36 |
| DW450 | *Gobiobotia meridionalis* | *Gobiobotia meridionalis* | JN003344 | 99.65 | *Gobiobotia naktongensis* | NC020464 | 89.36 |
| DW452 | *Gobiobotia meridionalis* | *Gobiobotia meridionalis* | JN003344 | 99.65 | *Gobiobotia naktongensis* | NC020464 | 89.36 |
| DW455 | *Gobiobotia meridionalis* | *Gobiobotia meridionalis* | JN003344 | 99.65 | *Gobiobotia naktongensis* | NC020464 | 89.36 |
| DW459 | *Gobiobotia meridionalis* | *Gobiobotia meridionalis* | JN003344 | 99.65 | *Gobiobotia naktongensis* | NC020464 | 89.36 |
| DW460 | *Gobiobotia meridionalis* | *Gobiobotia meridionalis* | JN003344 | 99.65 | *Gobiobotia naktongensis* | NC020464 | 89.36 |
| DW461 | *Gobiobotia meridionalis* | *Gobiobotia meridionalis* | JN003344 | 99.65 | *Gobiobotia naktongensis* | NC020464 | 89.36 |
| DW469 | *Gobiobotia meridionalis* | *Gobiobotia meridionalis* | JN003344 | 99.64 | *Gobiobotia naktongensis* | NC020464 | 89.52 |
| DW471 | *Gobiobotia meridionalis* | *Gobiobotia meridionalis* | JN003344 | 100 | *Gobiobotia filifer* | MK834305 | 89.36 |
| DW472 | *Gobiobotia meridionalis* | *Gobiobotia meridionalis* | JN003344 | 99.65 | *Gobiobotia naktongensis* | NC020464 | 89.36 |
| DW474 | *Gobiobotia meridionalis* | *Gobiobotia meridionalis* | JN003344 | 99.65 | *Gobiobotia naktongensis* | NC020464 | 89.36 |
| DW475 | *Gobiobotia meridionalis* | *Gobiobotia meridionalis* | JN003344 | 99.65 | *Gobiobotia naktongensis* | NC020464 | 89.36 |
| DW476 | *Gobiobotia meridionalis* | *Gobiobotia meridionalis* | JN003344 | 99.65 | *Gobiobotia naktongensis* | NC020464 | 89.36 |
| DW484 | *Gobiobotia meridionalis* | *Gobiobotia meridionalis* | JN003344 | 99.82 | *Gobiobotia filifer* | MK834305 | 89.18 |
| DW486 | *Gobiobotia meridionalis* | *Gobiobotia meridionalis* | JN003344 | 99.82 | *Gobiobotia filifer* | MK834305 | 89.36 |
| DW487 | *Gobiobotia meridionalis* | *Gobiobotia meridionalis* | JN003344 | 99.65 | *Gobiobotia naktongensis* | NC020464 | 89.36 |
| DW488 | *Gobiobotia meridionalis* | *Gobiobotia meridionalis* | JN003344 | 99.47 | *Gobiobotia naktongensis* | NC020464 | 89.54 |
| DW494 | *Gobiobotia meridionalis* | *Gobiobotia meridionalis* | JN003344 | 99.65 | *Gobiobotia naktongensis* | NC020464 | 89.36 |
| DW495 | *Gobiobotia meridionalis* | *Gobiobotia meridionalis* | JN003344 | 99.65 | *Gobiobotia naktongensis* | NC020464 | 89.36 |
| DW498 | *Gobiobotia meridionalis* | *Gobiobotia meridionalis* | JN003344 | 99.65 | *Gobiobotia naktongensis* | NC020464 | 89.36 |
| DW5 | *Pseudohemiculter dispar* | *Pseudohemiculter dispar* | Unpublished | 100 | Hemiculterella sauvagei | Unpublished | 91.94 |
| DW500 | *Gobiobotia meridionalis* | *Gobiobotia meridionalis* | JN003344 | 99.65 | *Gobiobotia naktongensis* | NC020464 | 89.36 |
| DW504 | *Gobiobotia meridionalis* | *Gobiobotia meridionalis* | JN003344 | 99.64 | *Gobiobotia filifer* | MK834305 | 89.13 |
| DW512 | *Gobiobotia meridionalis* | *Gobiobotia meridionalis* | JN003344 | 99.65 | *Gobiobotia naktongensis* | NC020464 | 89.36 |
| DW533 | *Gobiobotia meridionalis* | *Gobiobotia meridionalis* | JN003344 | 99.65 | *Gobiobotia naktongensis* | NC020464 | 89.36 |
| DW539 | *Gobiobotia meridionalis* | *Gobiobotia meridionalis* | JN003344 | 99.65 | *Gobiobotia naktongensis* | NC020464 | 89.36 |
| DW608 | *Pseudohemiculter dispar* | *Pseudohemiculter dispar* | Unpublished | 99.82 | *Hemiculterella sauvagei* | Unpublished | 91.53 |
| DW615 | *Sinibotia robusta* | *Sinibotia robusta* | MH027670 | 100 | *Sinibotia reevesae* | MH027676 | 91.67 |
| *DW616* | NA | NA | NA | NA | NA | NA | NA |
| DW617 | *Sinibotia robusta* | *Sinibotia robusta* | MH027668 | 99.82 | *Sinibotia superciliaris* | KC871100 | 91.83 |
| DW618 | *Squaliobarbus curriculus* | *Squaliobarbus curriculus* | KP769815 | 100 | *Elopichthys bambusa* | NC024834 | 94.68 |
| DW619 | *Sinibotia robusta* | *Sinibotia robusta* | KT374012 | 100 | *Sinibotia reevesae* | MH027676 | 91.84 |
| *DW620* | *Xenocypris* sp | *Xenocypris macrolepis* | Unpublished | 100 | *Xenocypris davidi* | Unpublished | 100 |
| DW621 | *Garra orientalis* | *Garra orientalis* | JQ864602 | 99.47 | *Garra cyrano* | MK116331 | 95.59 |
| DW622 | *Sinibotia robusta* | *Sinibotia robusta* | MH027668 | 99.82 | *Sinibotia superciliaris* | KC871100 | 91.83 |
| DW623 | *Sinibotia robusta* | *Sinibotia robusta* | MH027667 | 100 | *Sinibotia reevesae* | MH027676 | 92.2 |
| DW624 | *Sinibotia robusta* | *Sinibotia robusta* | KT374012 | 99.82 | *Sinibotia reevesae* | MH027676 | 92.02 |
| DW625 | *Xenocypris* spp | *Xenocypris macrolepis* | Unpublished | 100 | *Xenocypris davidi* | Unpublished | 100 |
| DW626 | *Sinibotia robusta* | *Sinibotia robusta* | MH027667 | 100 | *Sinibotia reevesae* | MH027676 | 92.2 |
| DW627 | *Sinibotia robusta* | *Sinibotia robusta* | MH027670 | 100 | *Sinibotia reevesae* | MH027676 | 91.67 |
| DW628 | *Garra orientalis* | *Garra orientalis* | JQ864602 | 100 | *Garra cyrano* | MK116331 | 95.79 |
| DW629 | *Sinibotia robusta* | *Sinibotia robusta* | MH027665 | 100 | *Sinibotia reevesae* | MH027676 | 92.02 |
| DW630 | *Garra orientalis* | *Garra orientalis* | JQ864602 | 100 | *Garra cyrano* | MK116331 | 95.79 |
| DW631 | *Sinibotia robusta* | *Sinibotia robusta* | KT374012 | 99.82 | *Sinibotia reevesae* | MH027676 | 92.02 |
| DW632 | *Sinibotia robusta* | *Sinibotia robusta* | KT374012 | 100 | *Sinibotia reevesae* | MH027676 | 91.84 |
| **DW633** | *Ctenopharyngodon idella* | *Ctenopharyngodon idella* | JN988835 | 100 | NA | NA | NA |
| DW634 | *Garra orientalis* | *Garra orientalis* | JQ864602 | 100 | *Garra cyrano* | MK116331 | 95.79 |
| DW635 | *Garra orientalis* | *Garra orientalis* | JQ864602 | 100 | *Garra cyrano* | MK116331 | 95.79 |
| **DW636** | *Ctenopharyngodon idella* | *Ctenopharyngodon idella* | JN988835 | 100 | NA | NA | NA |
| DW638 | *Garra orientalis* | *Garra orientalis* | NC021935 | 99.82 | *Garra cyrano* | MK116331 | 95.79 |
| DW639 | *Garra orientalis* | *Garra orientalis* | KX983931 | 100 | *Garra cyrano* | MK116331 | 95.58 |
| DW640 | *Garra orientalis* | *Garra orientalis* | KX983931 | 99.82 | *Garra cyrano* | MK116331 | 95.79 |
| *DW645* | NA | NA | NA | NA | NA | NA | NA |
| DW646 | *Garra orientalis* | *Garra orientalis* | JQ864602 | 100 | *Garra cyrano* | MK116331 | 95.79 |
| DW647 | *Sinibotia robusta* | *Sinibotia robusta* | KT374012 | 100 | *Sinibotia reevesae* | MH027676 | 91.84 |
| DW653 | *Garra orientalis* | *Garra orientalis* | JQ864602 | 100 | *Garra cyrano* | MK116331 | 95.79 |
| DW654 | *Garra orientalis* | *Garra orientalis* | JQ864602 | 100 | *Garra cyrano* | MK116331 | 95.79 |
| *DW655* | NA | NA | NA | NA | NA | NA | NA |
| DW661 | *Squalidus argentatus* | *Squalidus argentatus* | Unpublished | 100 | *Squalidus chankaensis* | Unpublished | 98.76 |
| DW663 | *Pseudohemiculter dispar* | *Pseudohemiculter dispar* | Unpublished | 100 | *Hemiculterella sauvagei* | Unpublished | 91.76 |
| DW664 | *Pseudohemiculter dispar* | *Pseudohemiculter dispar* | Unpublished | 100 | *Hemiculterella sauvagei* | Unpublished | 91.76 |
| DW665 | *Pseudohemiculter dispar* | *Pseudohemiculter dispar* | Unpublished | 100 | *Hemiculterella sauvagei* | Unpublished | 91.76 |
| DW666 | *Pseudohemiculter dispar* | *Pseudohemiculter dispar* | Unpublished | 100 | *Hemiculterella sauvagei* | Unpublished | 91.76 |
| DW667 | *Pseudohemiculter dispar* | *Pseudohemiculter dispar* | Unpublished | 100 | *Hemiculterella sauvagei* | Unpublished | 91.76 |
| DW669 | *Pseudohemiculter dispar* | *Pseudohemiculter dispar* | Unpublished | 100 | *Hemiculterella sauvagei* | Unpublished | 91.76 |
| DW670 | *Pseudohemiculter dispar* | *Pseudohemiculter dispar* | Unpublished | 100 | *Hemiculterella sauvagei* | Unpublished | 91.76 |
| DW671 | *Pseudohemiculter dispar* | *Pseudohemiculter dispar* | Unpublished | 100 | *Hemiculterella sauvagei* | Unpublished | 91.76 |
| DW672 | *Pseudohemiculter dispar* | *Pseudohemiculter dispar* | Unpublished | 100 | *Hemiculterella sauvagei* | Unpublished | 91.76 |
| DW673 | *Pseudohemiculter dispar* | *Pseudohemiculter dispar* | Unpublished | 100 | *Hemiculterella sauvagei* | Unpublished | 91.76 |
| DW674 | *Pseudohemiculter dispar* | *Pseudohemiculter dispar* | Unpublished | 100 | *Hemiculterella sauvagei* | Unpublished | 91.76 |
| DW675 | *Pseudohemiculter dispar* | *Pseudohemiculter dispar* | Unpublished | 100 | *Hemiculterella sauvagei* | Unpublished | 91.76 |
| DW676 | *Pseudohemiculter dispar* | *Pseudohemiculter dispar* | Unpublished | 99.82 | *Hemiculterella sauvagei* | Unpublished | 92.11 |
| DW677 | *Pseudohemiculter dispar* | *Pseudohemiculter dispar* | Unpublished | 100 | *Hemiculterella sauvagei* | Unpublished | 91.76 |
| DW678 | *Pseudohemiculter dispar* | *Pseudohemiculter dispar* | Unpublished | 100 | *Hemiculterella sauvagei* | Unpublished | 91.76 |
| DW679 | *Pseudohemiculter dispar* | *Pseudohemiculter dispar* | Unpublished | 100 | *Hemiculterella sauvagei* | Unpublished | 91.76 |
| DW680 | *Pseudohemiculter dispar* | *Pseudohemiculter dispar* | Unpublished | 100 | *Hemiculterella sauvagei* | Unpublished | 91.76 |
| DW681 | *Pseudohemiculter dispar* | *Pseudohemiculter dispar* | Unpublished | 100 | *Hemiculterella sauvagei* | Unpublished | 91.94 |
| DW683 | *Pseudohemiculter dispar* | *Pseudohemiculter dispar* | Unpublished | 100 | *Hemiculterella sauvagei* | Unpublished | 91.76 |
| DW684 | *Pseudohemiculter dispar* | *Pseudohemiculter dispar* | Unpublished | 100 | *Hemiculterella sauvagei* | Unpublished | 91.76 |
| DW685 | *Pseudohemiculter dispar* | *Pseudohemiculter dispar* | Unpublished | 100 | *Hemiculterella sauvagei* | Unpublished | 91.76 |
| DW686 | *Pseudohemiculter dispar* | *Pseudohemiculter dispar* | Unpublished | 100 | *Hemiculterella sauvagei* | Unpublished | 91.76 |
| DW687 | *Pseudohemiculter dispar* | *Pseudohemiculter dispar* | Unpublished | 100 | *Hemiculterella sauvagei* | Unpublished | 91.94 |
| DW688 | *Pseudohemiculter dispar* | *Pseudohemiculter dispar* | Unpublished | 100 | *Hemiculterella sauvagei* | Unpublished | 91.76 |
| DW690 | *Pseudohemiculter dispar* | *Pseudohemiculter dispar* | Unpublished | 100 | *Hemiculterella sauvagei* | Unpublished | 91.76 |
| DW691 | *Pseudohemiculter dispar* | *Pseudohemiculter dispar* | Unpublished | 100 | *Hemiculterella sauvagei* | Unpublished | 91.94 |
| DW692 | *Pseudohemiculter dispar* | *Pseudohemiculter dispar* | Unpublished | 100 | *Hemiculterella sauvagei* | Unpublished | 91.94 |
| DW695 | *Pseudohemiculter dispar* | *Pseudohemiculter dispar* | Unpublished | 100 | *Hemiculterella sauvagei* | Unpublished | 91.76 |
| DW696 | *Pseudohemiculter dispar* | *Pseudohemiculter dispar* | Unpublished | 100 | *Hemiculterella sauvagei* | Unpublished | 91.76 |
| DW7 | *Pseudohemiculter dispar* | *Pseudohemiculter dispar* | Unpublished | 100 | *Hemiculterella sauvagei* | Unpublished | 91.76 |
| DW701 | *Sinibotia pulchra* | *Sinibotia pulchra* | NC033950 | 100 | *Sinibotia superciliaris* | MH027682 | 90.78 |
| DW733 | *Pseudohemiculter dispar* | *Pseudohemiculter dispar* | Unpublished | 99.47 | *Hemiculterella sauvagei* | Unpublished | 92.02 |
| DW734 | *Pseudohemiculter dispar* | *Pseudohemiculter dispar* | Unpublished | 100 | *Hemiculterella sauvagei* | Unpublished | 91.76 |
| **DW735** | *Squalidus argentatus* | *Squalidus argentatus* | KR862242 | 100 | NA | NA | NA |
| DW737 | *Pseudohemiculter dispar* | *Pseudohemiculter dispar* | Unpublished | 100 | *Hemiculterella sauvagei* | Unpublished | 91.76 |
| DW738 | *Pseudohemiculter dispar* | *Pseudohemiculter dispar* | Unpublished | 100 | *Hemiculterella sauvagei* | Unpublished | 91.76 |
| DW739 | *Pseudohemiculter dispar* | *Pseudohemiculter dispar* | Unpublished | 100 | *Hemiculterella sauvagei* | Unpublished | 91.76 |
| DW741 | *Pseudohemiculter dispar* | *Pseudohemiculter dispar* | Unpublished | 100 | *Hemiculterella sauvagei* | Unpublished | 91.76 |
| DW742 | *Pseudohemiculter dispar* | *Pseudohemiculter dispar* | Unpublished | 99.82 | *Hemiculterella sauvagei* | Unpublished | 91.58 |
| DW743 | *Pseudohemiculter dispar* | *Pseudohemiculter dispar* | Unpublished | 100 | *Hemiculterella sauvagei* | Unpublished | 91.94 |
| DW744 | *Pseudohemiculter dispar* | *Pseudohemiculter dispar* | Unpublished | 99.82 | *Hemiculterella sauvagei* | Unpublished | 91.58 |
| DW745 | *Pseudohemiculter dispar* | *Pseudohemiculter dispar* | Unpublished | 100 | *Hemiculterella sauvagei* | Unpublished | 91.76 |
| DW746 | *Pseudohemiculter dispar* | *Pseudohemiculter dispar* | Unpublished | 100 | *Hemiculterella sauvagei* | Unpublished | 91.76 |
| DW747 | *Pseudohemiculter dispar* | *Pseudohemiculter dispar* | Unpublished | 100 | *Hemiculterella sauvagei* | Unpublished | 91.94 |
| DW748 | *Pseudohemiculter dispar* | *Pseudohemiculter dispar* | Unpublished | 100 | *Hemiculterella sauvagei* | Unpublished | 91.76 |
| DW752 | *Pseudohemiculter dispar* | *Pseudohemiculter dispar* | Unpublished | 100 | *Hemiculterella sauvagei* | Unpublished | 91.76 |
| DW755 | *Squalidus argentatus* | *Squalidus argentatus* | Unpublished | 99.65 | *Squalidus chankaensis* | Unpublished | 98.58 |
| DW757 | *Pseudohemiculter dispar* | *Pseudohemiculter dispar* | Unpublished | 100 | *Hemiculterella sauvagei* | Unpublished | 91.76 |
| DW758 | *Pseudohemiculter dispar* | *Pseudohemiculter dispar* | Unpublished | 100 | *Hemiculterella sauvagei* | Unpublished | 91.76 |
| DW759 | *Squalidus argentatus* | *Squalidus argentatus* | Unpublished | 99.65 | *Squalidus chankaensis* | Unpublished | 98.05 |
| DW760 | *Squalidus argentatus* | *Squalidus argentatus* | Unpublished | 100 | *Squalidus chankaensis* | Unpublished | 98.76 |
| DW761 | *Pseudohemiculter dispar* | *Pseudohemiculter dispar* | Unpublished | 100 | *Hemiculterella sauvagei* | Unpublished | 91.76 |
| DW762 | *Pseudohemiculter dispar* | *Pseudohemiculter dispar* | Unpublished | 100 | *Hemiculterella sauvagei* | Unpublished | 91.76 |
| DW763 | *Pseudohemiculter dispar* | *Pseudohemiculter dispar* | Unpublished | 100 | *Hemiculterella sauvagei* | Unpublished | 91.76 |
| DW766 | *Pseudohemiculter dispar* | *Pseudohemiculter dispar* | Unpublished | 100 | *Hemiculterella sauvagei* | Unpublished | 91.76 |
| DW788 | *Squalidus argentatus* | *Squalidus argentatus* | Unpublished | 99.82 | *Squalidus chankaensis* | Unpublished | 98.75 |
| DW789 | *Pseudohemiculter dispar* | *Pseudohemiculter dispar* | Unpublished | 100 | *Hemiculterella sauvagei* | Unpublished | 91.76 |
| DW790 | *Pseudohemiculter dispar* | *Pseudohemiculter dispar* | Unpublished | 100 | *Hemiculterella sauvagei* | Unpublished | 91.76 |
| DW791 | *Pseudohemiculter dispar* | *Pseudohemiculter dispar* | Unpublished | 100 | *Hemiculterella sauvagei* | Unpublished | 91.76 |
| DW792 | *Pseudohemiculter dispar* | *Pseudohemiculter dispar* | Unpublished | 100 | *Hemiculterella sauvagei* | Unpublished | 91.76 |
| DW794 | *Pseudohemiculter dispar* | *Pseudohemiculter dispar* | Unpublished | 99.82 | *Hemiculterella sauvagei* | Unpublished | 92.11 |
| DW795 | *Pseudohemiculter dispar* | *Pseudohemiculter dispar* | Unpublished | 100 | *Hemiculterella sauvagei* | Unpublished | 91.58 |
| DW796 | *Pseudohemiculter dispar* | *Pseudohemiculter dispar* | Unpublished | 100 | *Hemiculterella sauvagei* | Unpublished | 91.94 |
| DW797 | *Pseudohemiculter dispar* | *Pseudohemiculter dispar* | Unpublished | 100 | *Hemiculterella sauvagei* | Unpublished | 91.76 |
| DW798 | *Pseudohemiculter dispar* | *Pseudohemiculter dispar* | Unpublished | 100 | *Hemiculterella sauvagei* | Unpublished | 91.76 |
| DW8 | *Pseudohemiculter dispar* | *Pseudohemiculter dispar* | Unpublished | 100 | *Hemiculterella sauvagei* | Unpublished | 91.76 |
| DW802 | *Pseudohemiculter dispar* | *Pseudohemiculter dispar* | Unpublished | 100 | *Hemiculterella sauvagei* | Unpublished | 91.76 |
| DW804 | *Pseudohemiculter dispar* | *Pseudohemiculter dispar* | Unpublished | 100 | *Hemiculterella sauvagei* | Unpublished | 91.76 |
| DW805 | *Pseudohemiculter dispar* | *Pseudohemiculter dispar* | Unpublished | 100 | *Hemiculterella sauvagei* | Unpublished | 91.76 |
| DW806 | *Pseudohemiculter dispar* | *Pseudohemiculter dispar* | Unpublished | 100 | *Hemiculterella sauvagei* | Unpublished | 91.76 |
| DW807 | *Pseudohemiculter dispar* | *Pseudohemiculter dispar* | Unpublished | 100 | *Hemiculterella sauvagei* | Unpublished | 91.76 |
| DW808 | *Pseudohemiculter dispar* | *Pseudohemiculter dispar* | Unpublished | 99.82 | *Hemiculterella sauvagei* | Unpublished | 92.11 |
| DW809 | *Pseudohemiculter dispar* | *Pseudohemiculter dispar* | Unpublished | 100 | *Hemiculterella sauvagei* | Unpublished | 91.76 |
| DW810 | *Gobiobotia meridionalis* | *Gobiobotia meridionalis* | JN003344 | 99.65 | *Gobiobotia naktongensis* | NC020464 | 89.36 |
| DW812 | *Pseudohemiculter dispar* | *Pseudohemiculter dispar* | Unpublished | 100 | *Hemiculterella sauvagei* | Unpublished | 91.76 |
| DW812 | *Pseudohemiculter dispar* | *Pseudohemiculter dispar* | Unpublished | 100 | *Hemiculterella sauvagei* | Unpublished | 91.76 |
| DW813 | *Pseudohemiculter dispar* | *Pseudohemiculter dispar* | Unpublished | 100 | *Hemiculterella sauvagei* | Unpublished | 91.76 |
| DW814 | *Pseudohemiculter dispar* | *Pseudohemiculter dispar* | Unpublished | 100 | *Hemiculterella sauvagei* | Unpublished | 91.76 |
| DW815 | *Pseudohemiculter dispar* | *Pseudohemiculter dispar* | Unpublished | 100 | *Hemiculterella sauvagei* | Unpublished | 91.94 |
| DW816 | *Pseudohemiculter dispar* | *Pseudohemiculter dispar* | Unpublished | 100 | *Hemiculterella sauvagei* | Unpublished | 91.76 |
| DW817 | *Pseudohemiculter dispar* | *Pseudohemiculter dispar* | Unpublished | 100 | *Hemiculterella sauvagei* | Unpublished | 91.76 |
| DW818 | *Pseudohemiculter dispar* | *Pseudohemiculter dispar* | Unpublished | 99.82 | *Hemiculterella sauvagei* | Unpublished | 91.58 |
| DW819 | *Pseudohemiculter dispar* | *Pseudohemiculter dispar* | Unpublished | 100 | *Hemiculterella sauvagei* | Unpublished | 91.76 |
| DW821 | *Pseudohemiculter dispar* | *Pseudohemiculter dispar* | Unpublished | 100 | *Hemiculterella sauvagei* | Unpublished | 91.76 |
| DW825 | *Pseudohemiculter dispar* | *Pseudohemiculter dispar* | Unpublished | 99.82 | *Hemiculterella sauvagei* | Unpublished | 91.58 |
| DW827 | *Pseudohemiculter dispar* | *Pseudohemiculter dispar* | Unpublished | 100 | *Hemiculterella sauvagei* | Unpublished | 91.76 |
| DW828 | *Pseudohemiculter dispar* | *Pseudohemiculter dispar* | Unpublished | 100 | *Hemiculterella sauvagei* | Unpublished | 91.76 |
| DW829 | *Pseudohemiculter dispar* | *Pseudohemiculter dispar* | Unpublished | 100 | *Hemiculterella sauvagei* | Unpublished | 91.76 |
| DW830 | *Pseudohemiculter dispar* | *Pseudohemiculter dispar* | Unpublished | 100 | *Hemiculterella sauvagei* | Unpublished | 91.76 |
| DW831 | *Pseudohemiculter dispar* | *Pseudohemiculter dispar* | Unpublished | 100 | *Hemiculterella sauvagei* | Unpublished | 91.76 |
| DW832 | *Pseudohemiculter dispar* | *Pseudohemiculter dispar* | Unpublished | 100 | *Hemiculterella sauvagei* | Unpublished | 91.76 |
| DW838 | *Pseudohemiculter dispar* | *Pseudohemiculter dispar* | Unpublished | 100 | *Hemiculterella sauvagei* | Unpublished | 91.76 |
| DW839 | *Squalidus argentatus* | *Squalidus argentatus* | Unpublished | 99.82 | *Squalidus chankaensis* | Unpublished | 98.75 |
| DW840 | *Pseudohemiculter dispar* | *Pseudohemiculter dispar* | Unpublished | 100 | *Hemiculterella sauvagei* | Unpublished | 91.76 |
| DW841 | *Pseudohemiculter dispar* | *Pseudohemiculter dispar* | Unpublished | 99.64 | *Hemiculterella sauvagei* | Unpublished | 91.22 |
| DW842 | *Pseudohemiculter dispar* | *Pseudohemiculter dispar* | Unpublished | 100 | *Hemiculterella sauvagei* | Unpublished | 91.76 |
| DW844 | *Pseudohemiculter dispar* | *Pseudohemiculter dispar* | Unpublished | 100 | *Hemiculterella sauvagei* | Unpublished | 91.76 |
| **DW851** | *Squalidus argentatus* | *Squalidus argentatus* | KR862242 | 100 | NA | NA | NA |
| DW854 | *Pseudohemiculter dispar* | *Pseudohemiculter dispar* | Unpublished | 100 | *Hemiculterella sauvagei* | Unpublished | 91.94 |
| DW857 | *Pseudohemiculter dispar* | *Pseudohemiculter dispar* | Unpublished | 100 | *Hemiculterella sauvagei* | Unpublished | 91.76 |
| DW876 | *Gobiobotia meridionalis* | *Gobiobotia meridionalis* | JN003344 | 99.65 | *Gobiobotia naktongensis* | NC020464 | 89.36 |
| DW9 | *Pseudohemiculter dispar* | *Pseudohemiculter dispar* | Unpublished | 100 | *Hemiculterella sauvagei* | Unpublished | 91.76 |
| DW916 | *Gobiobotia meridionalis* | *Gobiobotia meridionalis* | JN003344 | 99.64 | *Gobiobotia naktongensis* | NC020464 | 89.3 |
| DW927 | *Gobiobotia meridionalis* | *Gobiobotia meridionalis* | JN003344 | 99.65 | *Gobiobotia naktongensis* | NC020464 | 89.36 |
| DW928 | *Pseudohemiculter dispar* | *Pseudohemiculter dispar* | Unpublished | 100 | *Hemiculterella sauvagei* | Unpublished | 91.76 |
| DW929 | *Squalidus argentatus* | *Squalidus argentatus* | Unpublished | 100 | *Squalidus chankaensis* | Unpublished | 98.76 |
| DW931 | *Gobiobotia meridionalis* | *Gobiobotia meridionalis* | JN003344 | 100 | *Gobiobotia filifer* | MK834305 | 89.36 |
| DW932 | *Sinibotia pulchra* | *Sinibotia pulchra* | MH027663 | 100 | *Sinibotia superciliaris* | MH027682 | 90.6 |
| DW933 | *Gobiobotia meridionalis* | *Gobiobotia meridionalis* | JN003344 | 99.65 | *Gobiobotia naktongensis* | NC020464 | 89.36 |
| DW934 | *Pseudohemiculter dispar* | *Pseudohemiculter dispar* | Unpublished | 100 | *Hemiculterella sauvagei* | Unpublished | 91.76 |
| DW935 | *Pseudohemiculter dispar* | *Pseudohemiculter dispar* | Unpublished | 100 | *Hemiculterella sauvagei* | Unpublished | 91.76 |
| DW936 | *Pseudohemiculter dispar* | *Pseudohemiculter dispar* | Unpublished | 100 | *Hemiculterella sauvagei* | Unpublished | 91.76 |
| DW937 | *Pseudohemiculter dispar* | *Pseudohemiculter dispar* | Unpublished | 100 | *Hemiculterella sauvagei* | Unpublished | 91.76 |
| DW938 | *Pseudohemiculter dispar* | *Pseudohemiculter dispar* | Unpublished | 100 | *Hemiculterella sauvagei* | Unpublished | 91.76 |
| DW939 | *Pseudohemiculter dispar* | *Pseudohemiculter dispar* | Unpublished | 100 | *Hemiculterella sauvagei* | Unpublished | 91.76 |
| DW940 | *Pseudohemiculter dispar* | *Pseudohemiculter dispar* | Unpublished | 100 | *Hemiculterella sauvagei* | Unpublished | 91.76 |
| DW941 | *Pseudohemiculter dispar* | *Pseudohemiculter dispar* | Unpublished | 100 | *Hemiculterella sauvagei* | Unpublished | 91.94 |
| DW942 | *Gobiobotia meridionalis* | *Gobiobotia meridionalis* | JN003344 | 100 | *Gobiobotia filifer* | MK834305 | 89.36 |
| DW943 | *Pseudohemiculter dispar* | *Pseudohemiculter dispar* | Unpublished | 100 | *Hemiculterella sauvagei* | Unpublished | 91.76 |
| DW944 | *Pseudohemiculter dispar* | *Pseudohemiculter dispar* | Unpublished | 100 | *Hemiculterella sauvagei* | Unpublished | 91.76 |
| DW945 | *Pseudohemiculter dispar* | *Pseudohemiculter dispar* | Unpublished | 100 | *Hemiculterella sauvagei* | Unpublished | 91.76 |
| DW946 | *Pseudohemiculter dispar* | *Pseudohemiculter dispar* | Unpublished | 100 | *Hemiculterella sauvagei* | Unpublished | 91.76 |
| DW948 | *Pseudohemiculter dispar* | *Pseudohemiculter dispar* | Unpublished | 100 | *Hemiculterella sauvagei* | Unpublished | 91.76 |
| DW949 | *Pseudohemiculter dispar* | *Pseudohemiculter dispar* | Unpublished | 100 | *Hemiculterella sauvagei* | Unpublished | 91.94 |
| DW950 | *Pseudohemiculter dispar* | *Pseudohemiculter dispar* | Unpublished | 100 | *Hemiculterella sauvagei* | Unpublished | 91.76 |
| DW951 | *Pseudohemiculter dispar* | *Pseudohemiculter dispar* | Unpublished | 100 | *Hemiculterella sauvagei* | Unpublished | 91.94 |
| DW952 | *Pseudohemiculter dispar* | *Pseudohemiculter dispar* | Unpublished | 100 | *Hemiculterella sauvagei* | Unpublished | 91.76 |
| DW954 | *Pseudohemiculter dispar* | *Pseudohemiculter dispar* | Unpublished | 100 | *Hemiculterella sauvagei* | Unpublished | 91.76 |
| DW955 | *Pseudohemiculter dispar* | *Pseudohemiculter dispar* | Unpublished | 100 | *Hemiculterella sauvagei* | Unpublished | 91.76 |
| DW956 | *Pseudohemiculter dispar* | *Pseudohemiculter dispar* | Unpublished | 100 | *Hemiculterella sauvagei* | Unpublished | 91.76 |
| DW957 | *Pseudohemiculter dispar* | *Pseudohemiculter dispar* | Unpublished | 100 | *Hemiculterella sauvagei* | Unpublished | 91.76 |
| DW958 | *Pseudohemiculter dispar* | *Pseudohemiculter dispar* | Unpublished | 100 | *Hemiculterella sauvagei* | Unpublished | 91.76 |
| DW961 | *Pseudohemiculter dispar* | *Pseudohemiculter dispar* | Unpublished | 100 | *Hemiculterella sauvagei* | Unpublished | 91.76 |
| DW962 | *Pseudohemiculter dispar* | *Pseudohemiculter dispar* | Unpublished | 100 | *Hemiculterella sauvagei* | Unpublished | 91.76 |
| DW964 | *Pseudohemiculter dispar* | *Pseudohemiculter dispar* | Unpublished | 100 | *Hemiculterella sauvagei* | Unpublished | 91.76 |
| DW966 | *Pseudohemiculter dispar* | *Pseudohemiculter dispar* | Unpublished | 100 | *Hemiculterella sauvagei* | Unpublished | 91.76 |
| DW968 | *Pseudohemiculter dispar* | *Pseudohemiculter dispar* | Unpublished | 100 | *Hemiculterella sauvagei* | Unpublished | 91.76 |
| DW969 | *Pseudohemiculter dispar* | *Pseudohemiculter dispar* | Unpublished | 100 | *Hemiculterella sauvagei* | Unpublished | 91.76 |
| DW971 | *Pseudohemiculter dispar* | *Pseudohemiculter dispar* | Unpublished | 100 | *Hemiculterella sauvagei* | Unpublished | 91.94 |
| DW978 | *Pseudohemiculter dispar* | *Pseudohemiculter dispar* | Unpublished | 100 | *Hemiculterella sauvagei* | Unpublished | 91.76 |
| DW981 | *Gobiobotia meridionalis* | *Gobiobotia meridionalis* | JN003344 | 99.47 | *Gobiobotia naktongensis* | NC020464 | 89.54 |
| **DW983** | *Squalidus argentatus* | *Squalidus argentatus* | KR862242 | 100 | NA | NA | NA |
| DW984 | *Pseudohemiculter dispar* | *Pseudohemiculter dispar* | Unpublished | 100 | *Hemiculterella sauvagei* | Unpublished | 91.76 |
| DW985 | *Pseudohemiculter dispar* | *Pseudohemiculter dispar* | Unpublished | 100 | *Hemiculterella sauvagei* | Unpublished | 91.76 |
| DW986 | *Pseudohemiculter dispar* | *Pseudohemiculter dispar* | Unpublished | 100 | *Hemiculterella sauvagei* | Unpublished | 91.76 |
| *DWZY10* | *Siniperca* spp | *Siniperca scherzeri* | Unpublished | 100 | *Siniperca roulei* | NC024432 | 99.82 |
| *DWZY11* | *Siniperca* spp | *Siniperca scherzeri* | Unpublished | 100 | *Siniperca roulei* | NC024432 | 99.82 |
| *DWZY12* | NA | NA | NA | NA | NA | NA | NA |
| *DWZY13* | NA | NA | NA | NA | NA | NA | NA |
| *DWZY14* | *Siniperca* spp | *Siniperca scherzeri* | Unpublished | 100 | *Siniperca roulei* | NC024432 | 99.82 |
| DWZY15 | *Zacco platypus* | *Zacco platypus* | Unpublished | 100 | *Zacco acutipinnis* | NC028595 | 93.65 |
| DWZY17 | *Mastacembelus armatus* | *Mastacembelus armatus* | Unpublished | 100 | *Mastacembelus cf. favus* | KT944658 | 98.4 |
| DWZY2 | *Onychostoma gerlachi* | *Onychostoma gerlachi* | KY653684 | 100 | *Onychostoma meridionale* | AP011351 | 93.62 |
| *DWZY3* | *Siniperca* spp | *Siniperca scherzeri* | Unpublished | 100 | *Siniperca roulei* | NC024432 | 99.82 |
| DWZY4 | *Pseudohemiculter dispar* | *Pseudohemiculter dispar* | Unpublished | 100 | *Hemiculterella sauvagei* | Unpublished | 91.76 |
| DWZY5 | *Onychostoma gerlachi* | *Onychostoma gerlachi* | KY653684 | 100 | *Onychostoma meridionale* | AP011351 | 93.62 |
| DWZY6 | *Onychostoma gerlachi* | *Onychostoma gerlachi* | KY653684 | 100 | *Onychostoma meridionale* | AP011351 | 93.62 |
| DWZY7 | *Onychostoma gerlachi* | *Onychostoma gerlachi* | KJ994644 | 99.82 | *Onychostoma meridionale* | AP011351 | 93.44 |
| *DWZY8* | NA | NA | NA | NA | NA | NA | NA |
| *DWZY9* | *Rhinogobius* spp1 | *Rhinogobius leavelli* | AB988820 | 100 | *Rhinogobius maculafasciatus* | KU944927 | 99.45 |
| DWZY16 | *Onychostoma gerlachi* | *Onychostoma gerlachi* | KY653684 | 100 | *Onychostoma meridionale* | AP011351 | 93.62 |
| DWZY18 | *Onychostoma gerlachi* | *Onychostoma gerlachi* | KY653684 | 100 | *Onychostoma meridionale* | AP011351 | 93.62 |
