# Supplementary material for: DNA barcoding reveals the temporal community composition of drifting fish eggs in the lower Hongshui River, China
Source: Ecol Evol. 2021 Jul 22;11(16):11507–14. doi: 10.1002/ece3.7943 (PMC8366882; doi:10.1002/ece3.7943)
Supplement: Supplementary file 3 — Table S3 [file ECE3-11-11507-s001.docx]

| Sample ID | Final name | TOP1 | TOP2 | TOP3 | TOP4 | TOP5 |
| --- | --- | --- | --- | --- | --- | --- |
| DWZY9 | *Rhinogobius* spp1 | *Rhinogobius leavelli* (100) | *Rhinogobius cliffordpopei* (99.30) | *Rhinogobius cliffordpopei* (98.85) | *Rhinogobius cliffordpopei* (98.85) | *Rhinogobius cliffordpopei* (98.85) |
| DWZY8 | *Rhinogobius* spp2 | *Rhinogobius duospilus* (97.72) | *Rhinogobius virgigena* (96.66) | *Rhinogobius* sp. (96.72) | *Rhinogobius* sp. (96.53) | *Rhinogobius* sp. (96.53) |
| DWZY13 | *Rhinogobius* spp2 | *Rhinogobius duospilus* (97.72) | *Rhinogobius virgigena* (96.66) | *Rhinogobius* sp. (96.72) | *Rhinogobius* sp. (96.53) | *Rhinogobius* sp. (96.53) |
| DWZY3 | *Siniperca scherzeri* | *Siniperca scherzeri* (100) | *Siniperca scherzeri* (100) | *Siniperca scherzeri* (100) | *Siniperca scherzeri* (100) | *Siniperca scherzeri* (100) |
| DWZY10 | *Siniperca scherzeri* | *Siniperca scherzeri* (100) | *Siniperca scherzeri* (100) | *Siniperca scherzeri* (100) | *Siniperca scherzeri* (100) | *Siniperca scherzeri* (100) |
| DWZY11 | *Siniperca scherzeri* | *Siniperca scherzeri* (100) | *Siniperca scherzeri* (100) | *Siniperca scherzeri* (100) | *Siniperca scherzeri* (100) | *Siniperca scherzeri* (100) |
| DWZY14 | *Siniperca scherzeri* | *Siniperca scherzeri* (100) | *Siniperca scherzeri* (100) | *Siniperca scherzeri* (100) | *Siniperca scherzeri* (100) | *Siniperca scherzeri* (100) |
| DW620 | *Xenocypris* spp | *Xenocypris davidi* (98.95) | *Xenocypris davidi* (98.95) | *Xenocypris argentea* (98.95) | *Xenocypris argentea* (98.95) | *Xenocypris davidi* (98.95) |
| DW625 | *Xenocypris* spp | *Xenocypris davidi* (98.95) | *Xenocypris davidi* (98.95) | *Xenocypris argentea* (98.95) | *Xenocypris argentea* (98.95) | *Xenocypris davidi* (98.95) |
| DW645 | Unknown species 1 | *Balitora kwangsiensis* (92.97) | NA | NA | NA | NA |
| DW616 | Unknown species 1 | *Balitora kwangsiensis* (92.79) | NA | NA | NA | NA |
| DW655 | Unknown species 1 | *Balitora kwangsiensis* (92.62) | NA | NA | NA | NA |
| DWZY12 | Unknown species 2 | *Rhinogobius wuyanlingensis* (90.86) | NA | NA | NA | NA |
