# Supplementary material for: DNA barcoding reveals the temporal community composition of drifting fish eggs in the lower Hongshui River, China
Source: Ecol Evol. 2021 Jul 22;11(16):11507–14. doi: 10.1002/ece3.7943 (PMC8366882; doi:10.1002/ece3.7943)
Supplement: Supplementary file 4 — Table S4 [file ECE3-11-11507-s002.docx]

>*Xenocypris* sp_DW620

CTTCTCATTCGAGCCGAACTAAGTCAACCCGGATCACTTCTGGGCGATGACCAAATTTATAATGTTATTGTTACTGCCCATGCCTTCGTAATAATTTTCTTTATAGTAATACCAATTCTTATTGGAGGGTTTGGAAATTGACTCGTTCCACTAATAATTGGAGCGCCTGATATGGCATTCCCACGAATAAACAACATAAGCTTCTGACTTCTACCTCCTTCTTTCCTCCTGCTATTAGCCTCTTCCGGAGTCGAGGCCGGAGCTGGGACAGGATGAACAGTTTACCCGCCACTCGCAGGCAACCTTGCCCATGCTGGAGCATCCGTAGACCTAACAATTTTCTCACTTCACCTAGCAGGTGTATCATCAATTCTAGGGGCAATTAACTTCATCACTACAACTATTAACATGAAACCACCAGCCATTTCCCAATACCAAACACCTCTGTTCGTCTGAGCTGTACTTGTAACAGCCGTACTTCTTCTCCTATCACTACCAGTCCTAGCTGCCGGAATTACAATGCTCCTTACAGACCGAAATCTTAACACCACATTCTTCGACCCGGCAGG

>*Xenocypris* sp_DW625

CTTCTCATTCGAGCCGAACTAAGTCAACCCGGATCACTTCTGGGCGATGACCAAATTTATAATGTTATTGTTACTGCCCATGCCTTCGTAATAATTTTCTTTATAGTAATACCAATTCTTATTGGAGGGTTTGGAAATTGACTCGTTCCACTAATAATTGGAGCGCCTGATATGGCATTCCCACGAATAAACAACATAAGCTTCTGACTTCTACCTCCTTCTTTCCTCCTGCTATTAGCCTCTTCCGGAGTCGAGGCCGGAGCTGGGACAGGATGAACAGTTTACCCGCCACTCGCAGGCAACCTTGCCCATGCTGGAGCATCCGTAGACCTAACAATTTTCTCACTTCACCTAGCAGGTGTATCATCAATTCTAGGGGCAATTAACTTCATCACTACAACTATTAACATGAAACCACCAGCCATTTCCCAATACCAAACACCTCTGTTCGTCTGAGCTGTACTTGTAACAGCCGTACTTCTTCTCCTATCACTACCAGTCCTAGCTGCCGGAATTACAATGCTCCTTACAGACCGAAATCTTAACACCACATTCTTCGACCCGGCAGG

>*Rhinogobius* sp.1_DWZY9

CTCCTCATTCGAGCCGAGCTAAGCCAGCCCGGAGCCCTTCTGGGTGATGACCAGATTTACAATGTAATCGTAACAGCTCATGCTTTCGTAATAATTTTCTTTATAGTAATACCAATTATAATTGGAGGGTTTGGAAACTGACTAATTCCTCTGATGATCGGCGCTCCCGACATGGCTTTCCCCCGAATGAACAACATGAGCTTTTGACTCCTGCCCCCTTCCTTCTTACTCCTCCTGGCTTCTTCGGGAGTTGAAGCCGGGGCAGGTACCGGATGAACTGTCTACCCGCCCCTCGCCGGGAACCTCGCCCATGCCGGCGCCTCTGTTGACTTAACCATTTTCTCTCTCCACTTGGCAGGCATTTCCTCTATTCTAGGGGCCATTAACTTCATTACGACCATCCTAAATATGAAGCCCCCTGCAATCTCACAATACCAAACCCCCCTATTCGTGTGGGCTGTACTAATTACAGCTGTCCTTTTACTTCTTTCCCTCCCCGTTCTTGCCGCCGGCATTACAATGCTTCTTACAGACCGAAACCTAAACACGACCTTCTTTGACCCGGCAGG

>*Rhinogobius* sp.2_DWZY8

CTCCTTATTCGAGCCGAGCTTAGCCAGCCCGGAGCCCTTCTGGGCAATGACCAAATCTATAATGTTATCGTTACAGCTCATGCTTTCGTAATAATCTTCTTTATAGTAATACCAATTATGATTGGGGGTTTCGGAAACTGGCTTGTTCCCCTAATGATTGGTGCACCAGACATAGCCTTCCCTCGAATAAATAATATAAGCTTTTGACTTCTCCCCCCATCCTTCCTTCTTCTATTGGCTTCTTCGGGAGTTGAAGCCGGGGCAGGTACCGGATGAACTGTATATCCCCCACTAGCCGGGAATCTTGCCCATGCCGGCGCCTCTGTTGACTTAACCATCTTCTCCCTCCATCTGGCTGGTATTTCCTCCATCCTCGGGGCCATTAACTTCATCACAACTATTATTAACATGAAACCTCCTGCAATCTCACAATACCAAACTCCACTTTTCGTGTGAGCCGTCTTAATTACAGCCGTACTTCTACTCCTCTCACTTCCGGTTCTCGCTGCCGGTATTACAATGCTTCTCACAGACCGAAACCTAAATACGACCTTCTTTGATCCGGCAGG

>*Rhinogobius* sp.2_DWZY13

CTCCTTATTCGAGCCGAGCTTAGCCAGCCCGGAGCCCTTCTGGGCAATGACCAAATCTATAATGTTATCGTTACAGCTCATGCTTTCGTAATAATCTTCTTTATAGTAATACCAATTATGATTGGGGGTTTCGGAAACTGGCTTGTTCCCCTAATGATTGGTGCACCAGACATAGCCTTCCCTCGAATAAATAATATAAGCTTTTGACTTCTCCCCCCATCCTTCCTTCTTCTATTGGCTTCTTCGGGAGTTGAAGCCGGGGCAGGTACCGGATGAACTGTATATCCCCCACTAGCCGGGAATCTTGCCCATGCCGGCGCCTCTGTTGACTTAACCATCTTCTCCCTCCATCTGGCTGGTATTTCCTCCATCCTCGGGGCCATTAACTTCATCACAACTATTATTAACATGAAACCTCCTGCAATCTCACAATACCAAACTCCACTTTTCGTGTGAGCCGTCTTAATTACAGCCGTACTTCTACTCCTCTCACTTCCGGTTCTCGCTGCCGGTATTACAATGCTTCTCACAGACCGAAACCTAAATACGACCTTCTTTGATCCGGCAGG

>Unknown species 1_DW616

CTGCTAATCCGCGCTGAACTAAACCAACCCGGATCACTTCTTGGTGATGACCAAATTTATAATGTCATTGTTACTGCACACGCCTTTGTTATAATTTTCTTTATAGTAATGCCAATTCTTATTGGGGGATTTGGTAACTGACTAGTACCCCTAATAATTGGGGCCCCGGATATAGCATTCCCACGAATAAACAATATAAGCTTCTGACTCCTGCCACCATCCTTTCTGTTACTCCTAGCCTCATCTGGAGTAGAAGCCGGTGCAGGAACGGGATGAACTGTCTATCCACCACTGGCGGGCAACCTCGCCCACGCAGGAGCATCCGTAGACCTGACCATTTTCTCTCTGCACTTAGCTGGTGTGTCTTCTATTCTAGGAGCAATCAATTTTATTACCACAACTATCAATATAAAACCCCCAGCCATTTCCCAGTATCAAACACCACTATTTGTATGAGCCGTACTAGTAACAGCAGTTCTTCTACTGCTCTCACTACCCGTTCTAGCGGCTGGAATTACAATACTTTTAACAGACCGAAACTTAAACACAACATTCTTTGACCCAGCAGG

>Unknown species 1_DW645

CTGCTAATCCGCGCTGAACTAAACCAACCCGGATCACTTCTTGGTGATGACCAAATTTATAATGTCATTGTTACTGCACACGCCTTTGTTATAATTTTCTTTATAGTAATGCCAATTCTTATTGGGGGATTTGGTAACTGACTAGTACCCCTAATAATTGGGGCCCCGGATATAGCATTCCCACGAATAAACAATATAAGCTTCTGACTCCTGCCACCATCCTTTCTGTTACTCCTAGCCTCATCTGGAGTAGAAGCCGGTGCAGGAACGGGATGAACTGTCTATCCACCACTGGCGGGCAACCTCGCCCACGCAGGAGCATCCGTAGACCTGACCATTTTCTCTCTGCACTTAGCTGGTGTGTCTTCTATTCTAGGGGCAATCAATTTTATTACCACAACTATCAATATAAAACCCCCAGCCATTTCCCAGTATCAAACACCACTATTTGTATGAGCCGTACTAGTAACAGCAGTTCTTCTACTGCTCTCACTACCCGTTCTAGCGGCTGGAATTACAATACTTTTAACAGACCGAAACTTAAACACAACATTCTTTGACCCAGCAGG

>Unknown species 1_DW655

CTGCTAATCCGCGCTGAACTAAACCAACCCGGATCACTTCTTGGTGATGACCAAATTTATAATGTCATTGTTACTGCACACGCCTTTGTTATAATTTTCTTTATAGTAATGCCAATTCTTATTGGGGGATTTGGTAACTGACTAGTACCCCTAATAATTGGGGCCCCGGATATAGCATTCCCACGAATAAACAATATAAGCTTCTGACTCCTGCCACCATCCTTTCTGTTACTCCTAGCCTCATCTGGAGTAGAAGCCGGTGCAGGAACGGGATGAACTGTCTATCCACCACTGGCGGGCAACCTCGCCCACGCAGGAGCATCCGTAGACCTGACCATTTTCTCTCTGCACTTAGCTGGTGTGTCTTCTATTCTAGGAGCAATCAATTTTATTACCACAACTATCAATATAAAACCCCCAGCCATTTCCCAGTATCAAACACCACTATTTGTATGAGCCGTACTAGTAACAGCAGTTCTTCTACTGCTCTCACTACCAGTTCTAGCGGCTGGAATTACAATACTTTTAACAGACCGAAACTTAAACACAACATTCTTTGACCCAGCAGG

>Unknown species 2_DWZY12

CTCCTTATTCGAGCCGAACTAAGCCAGCCCGGAGCCCTTCTGGGCGATGACCAAATCTACAACGTTATCGTTACAGCTCATGCCTTCGTAATAATTTTCTTTATAGTAATACCAATCATGATTGGAGGATTCGGAAACTGGCTAGTCCCCCTGATAATTGGCGCACCAGACATGGCCTTCCCTCGAATGAACAACATAAGCTTCTGACTTCTGCCCCCCTCGTTCCTACTACTATTAGCTTCTTCAGGAGTTGAAGCGGGAGCAGGCACCGGATGAACTGTCTACCCCCCACTAGCCGGAAACCTCGCCCATGCCGGAGCCTCCGTTGACTTAACCATTTTCTCCCTTCATCTGGCTGGTATTTCCTCCATCCTTGGGGCCATTAACTTCATCACAACTATTATTAACATGAAGCCTCCTGCAATCTCACAATACCAGACCCCCCTTTTCGTGTGAGCAGTTCTCATTACAGCCGTACTCCTACTTCTCTCCCTTCCGGTTCTTGCTGCCGGCATCACAATACTTCTTACAGACCGGAACCTAAATACAACCTTCTTTGACCCGGCAGG
